# Operation model of a skew-symmetric split-crystal neutron interferometer

**DOI:** 10.1107/S1600576723010245

**Published:** 2024-02-01

**Authors:** Carlo P. Sasso, Giovanni Mana, Enrico Massa

**Affiliations:** a INRIM – Istituto Nazionale di Ricerca Metrologica, Strada delle cacce 91, 10135 Torino, Italy; b Dipartimento di Fisica, UNITO – Università di Torino, via Pietro Giuria 1, 10125 Torino, Italy; Oak Ridge National Laboratory, USA; North Carolina State University, USA

**Keywords:** split-crystal interferometry, neutron interferometry, X-ray interferometry, dynamical theory of X-ray diffraction, X-ray and neutron coherence

## Abstract

The observation of neutron interference using a triple Laue interferometer formed by two separate crystals opens the way to the construction and operation of skew-symmetric interferometers with extended arm separation and length. The specifications necessary for their successful operation are investigated here: most importantly, how the manufacturing tolerance and crystal alignments impact the interference visibility. In contrast with previous studies, both incoherent sources and the three-dimensional operation of the interferometer are considered. It is found that, with a Gaussian Schell model of an incoherent source, the integrated density of the particles leaving the interferometer is the same as that yielded by a coherent Gaussian source having a radius equal to the coherence length.

## Abstract 2

The observation of neutron interference using a triple Laue interferometer formed by two separate crystals opens the way to the construction and operation of skew-symmetric interferometers with extended arm separation and length. The specifications necessary for their successful operation are investigated here. In contrast with previous studies, both incoherent sources and the three-dimensional operation of the interferometer are considered.

## 1. Introduction

Since its first demonstrations by Bonse and Hart in 1965 (Bonse & Hart, 1965 ▸) and Rauch and collaborators in 1974 (Rauch *et al.*, 1974 ▸), perfect-crystal interferometry has been a powerful tool to perform phase-contrast imaging and metrology with X-rays and neutrons (Rauch & Werner, 2000 ▸; Tamasaku *et al.*, 2002 ▸; Momose, 2003 ▸; Klein, 2009 ▸; Pignol *et al.*, 2015 ▸; Massa *et al.*, 2020 ▸; Sponar *et al.*, 2021 ▸; Heacock *et al.*, 2021 ▸).

A proof-of-principle demonstration has shown that the alignment and operation of a split-crystal interferometer with the accuracy required for neutron interference are possible (Lemmel *et al.*, 2022 ▸). This prompts the design and operation of skew-symmetric interferometers operating with both X-rays and neutrons and having the potential of crystal separations up to the metre scale.

This work aims to understand the machining and alignment specifications necessary for the successful design, manufacture and operation of such a skew-symmetric split-crystal interferometer. Becker & Bonse (1974 ▸) observed unexpected and unexplained interference fringes associated with the relative tilt of split crystals having a period of 0.3 µrad, which might complicate the instrument’s operation. Windisch & Becker (1992 ▸) proposed a mirror crystal with twice the thickness of the splitter and the analyser to achieve fringe contrast also far from the perfect Bragg alignment of the split crystals.

On the basis of the formalism developed by Sasso *et al.* (2022 ▸), we here quantify the effects of the design parameters, machining tolerances and misalignments on the interferometer operation. In contrast to previous studies (Becker & Bonse, 1974 ▸; Bauspiess *et al.*, 1976 ▸; Bonse & Graeff, 1977 ▸; Bonse, 1988 ▸; Windisch & Becker, 1992 ▸; Mana & Vittone, 1997 ▸; Authier, 2001 ▸; Mana & Montanari, 2004 ▸), we consider the propagation, in three dimensions, of partially coherent X-rays and neutrons.

The paper is organized as follows. The dynamical theory of X-ray and neutron propagation in perfect crystals is recalled in Section 2. Section 3 gives the mathematical tools to propagate X-rays and neutrons through a split-crystal skew-symmetric interferometer by taking all the relevant degrees of freedom into account. A partially coherent source is modeled in Section 4. Sections 5 and 6 examine the interference of the waves leaving the interferometer, first assuming coherent illumination and then incoherent. The results of numerical and Monte Carlo simulations investigating the interferometer’s sensitivity to crystal thicknesses and misalignments and the manufacturing tolerances are given in Sections 7 and 8.

We have omitted the effects of gravity, the Coriolis force and interferometer accelerations (Bonse & Wroblewski, 1983 ▸, 1984 ▸). Taking them into account requires the quantum propagators of neutrons subjected to the gravitational and Coriolis forces in free space, and perfect crystals and crystal interferometers; these will be the subject of a forthcoming paper.

We carried out all the computations with the aid of *Mathematica* (Wolfram Research, 2021*a* ▸); the relevant notebook and a PDF rendering of the script are given as supporting information. To view and interact with the notebook, the reader may download the *Wolfram Player* free of charge (Wolfram Research, 2021*b* ▸).

## 2. Dynamical theory of diffraction

### 2.1. Crystal fields

**Figure 1:**
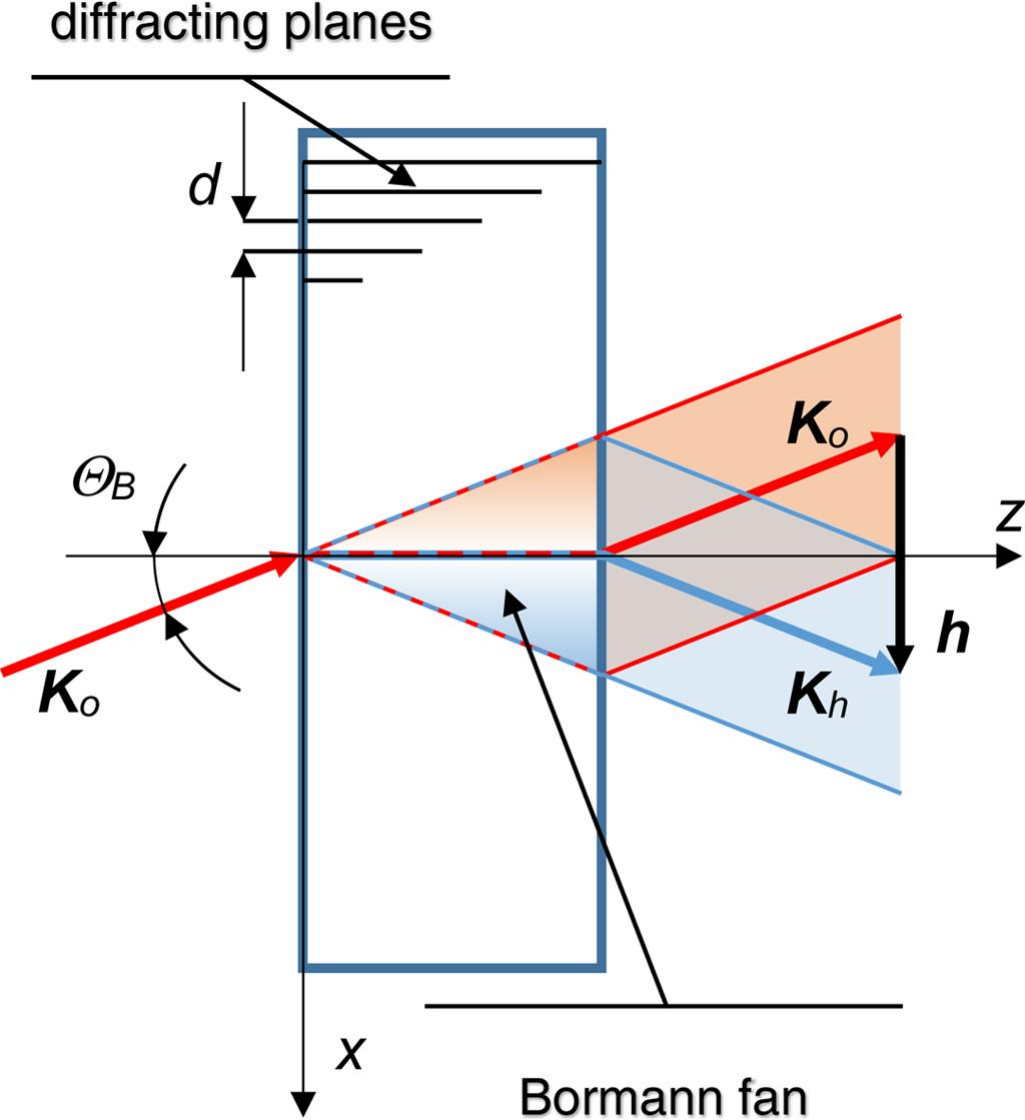
The incoming free-space *o* mode excites two guided modes within the crystal, which are linear superpositions of the *o* and *h* modes. The guided modes propagate parallel to the diffracting planes and spread into the Bormann fan. At the exit, they excite the free-space *o* and *h* modes, whose intensities depend on the crystal thickness (the dual of the impulse duration in atom interferometry). In a symmetrically cut crystal, the diffracting planes are orthogonal to the crystal surfaces.

As Fig. 1 ▸ shows, we assume symmetrically cut and plane-parallel crystals. The normal 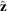 to the surfaces and the reciprocal vector 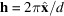 , where *d* is the spacing of the diffracting planes, define a reference frame. The origin is on the crystal surface and the *y* axis points up. The position vector **r** = 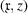 is split into the 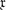 = (*x*, *y*) component (on the crystal surface) and the *z* component which determines the optical axis and plays the role of fictitious time.

Let us introduce the single-particle state, 

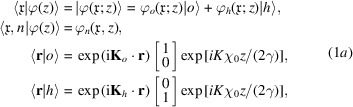

which belongs to the tensor product 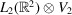 of the 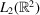 space of the square-integrable two-variable functions and the two-dimensional vector space *V*_2_. We use the 2 × 1 matrix representation of *V*_2_, 

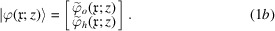

In (1*a* [Disp-formula fd1a]), γ = 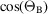 , where Θ_B_ is the Bragg angle, and 

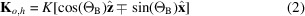

are the kinematic wavevectors satisfying the Bragg conditions **K**_*h*_ = **K**_*o*_ + **h** and |**K**_*o*_| = |**K**_*h*_| = *K* = 2π/λ, where λ is the wavelength of the incident radiation. We also consider a coplanar geometry, that is, **K**_*o*_, **K**_*h*_, **h** and 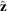 are in the same (horizontal) reflection plane.

To discuss X-ray and neutron interferometry together, the coefficients υ_*h*_ of the Fourier expansion of the periodic Fermi pseudo-potential (Rauch & Werner, 2000 ▸) are linked to the coefficients χ_*h*_ of the Fourier expansion of the crystal dielectric susceptibility (Stepanov, 2004 ▸) by setting χ_*h*_ = −υ_*h*_/*K* ^2^. Also, in the X-ray case, we consider only a polarization state that is parallel or orthogonal to the reflection plane.

We kept the effects of absorption, μ = Im(χ_0_)*K*, and refractive index, *n*_0_ − 1 = Re(χ_0_)/2, apart in the 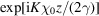 factor, where χ_0_ = 0 in a vacuum. In our analysis, this factor is irrelevant and will be omitted.

The χ_±*h*_ phases depend on the origin of the coordinate system in the unit cell; a translation **u** changes χ_±*h*_ according to 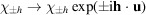 . We assume that χ(−*x*; *z*) = χ(*x*; *z*), so that χ_*h*_ = χ_−*h*_. Furthermore, since 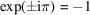 , the sign of χ_±*h*_ can be either positive or negative.

### 2.2. Free-space propagation

Neglecting gravity and the Coriolis force, free-space propagation is given by 

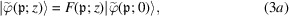

where (see Appendix *A* [App appa]) 

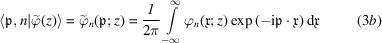

is the reciprocal-space representation of |ψ(*z*)〉, the resonance error 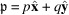 is the variable conjugate of 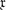 , 

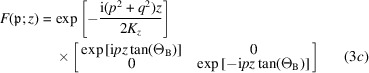

is the transfer matrix and 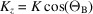 is the **K**_*o*,*h*_ component along the *z* axis.

### 2.3. Crystal diffraction

Still neglecting gravity and the Coriolis force, Laue diffraction in a symmetrically cut crystal is given by 

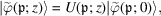

where, assuming that *x* = 0 is a symmetry plane of the crystal, the transfer matrix is 

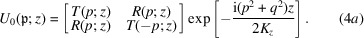

The reflection and transmission coefficients are 

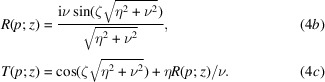

Here, ζ = π*z*/Λ_*e*_ is the dimensionless propagation distance, 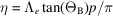 is the dimensionless resonance error, Λ_*e*_ = 2πγ/(*K*|χ_*h*_|) is the pendellösung length and ν = χ_*h*_/|χ_*h*_| is the χ_*h*_ phasor. If the symmetry plane of the crystal is *x* = *s*, then 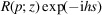 substitutes for 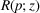 . A list of the main symbols used is given in Appendix *E* [App appe].

## 3. Interferometer operation

To increase the separation and length of the arms, a skew-symmetric interferometer is necessary (Kuetgens & Becker, 1998 ▸; Yoneyama *et al.*, 1999 ▸). It consists of two independent crystals that we denote I and II, each of which has two diffracting slabs protruding from the same base. In this configuration, the displacement of one crystal against the other does not affect the interference. They can be positioned far apart and the interferometer can be operated by tuning two angles, the mutual yaw and pitch angles of the split crystals.

**Figure 2:**
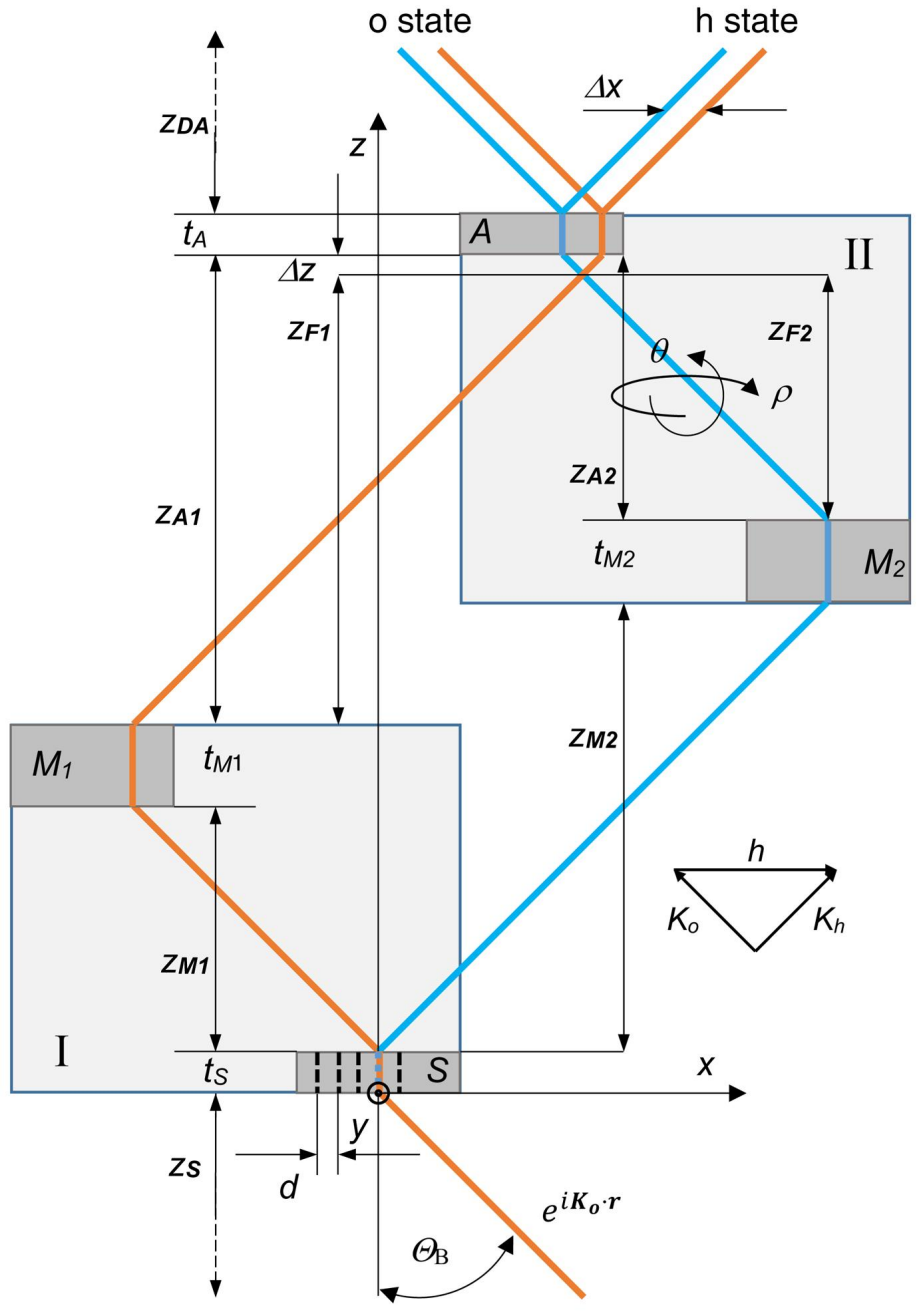
Skew-symmetric triple Laue interferometer with split crystals I and II (top view). The components are labelled as follows: *S* splitter, *M*_1_ and *M*_2_ mirrors, *A* analyser. Orange and blue rays indicate arms 1 and 2, respectively. The rays forward transmitted by the two mirrors have been omitted. The *x* axis is orthogonal to the diffracting planes. Θ_B_ is the Bragg angle and 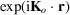 is the 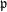 = 0 rad m^−1^ mode of the incoming single-particle wavefunction. ρ (mutual pitch angle) and θ (mutual yaw angle) are rotation angles about the *z* and *y* axes, respectively. The *y* axis points up.

Since the two crystals might be oriented and displaced differently (Figs. 2 ▸, 3 ▸ and 4 ▸), the representations of the neutron state and transfer matrix – equations (4*a* [Disp-formula fd4a]) and (1*a* [Disp-formula fd1a]), respectively – cannot be simultaneously used for both because the crystals’ kinematic vectors defined in (2) are different.

To express the single-particle state leaving crystal I on the basis of the kinematic wavevectors relevant to crystal II, we observe that the position vector **r**_I_ (as seen by crystal I and relative to the interferometer focus, which is the point in the reflection plane where the split particles are recombined) is seen from crystal II as 

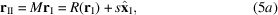

where 

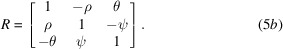

The yaw θ, pitch ρ and roll ψ angles are the rotation angles about the *y*, *z* and *x* axes, respectively. We introduced the displacement *s* along the *x* axis because it shifts the reference-frame origin versus the diffracting planes and it is not *ab initio* evident that it is irrelevant. In contrast, the *y* (vertical) and *z* (axial) translations are irrelevant and have been omitted.

From the viewpoint of crystal I, crystal II first rotates about the focal axis (see Figs. 2 ▸ and 4 ▸), 

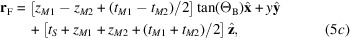

and then translates by 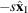 . From the vantage point of crystal I, crystal II counter-rotates and counter-translates.

The problem to be solved is constructing the unitary operator that implements the reference-frame transformation in the 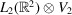 space. *Mutatis mutandis*, it is the same problem as constructing an operator that allows propagation of a polarized photon through a birefringent crystal whose transfer matrix is given in the basis of the crystal’s eigen­vectors, while the photon polarization state is given in a different basis.

The values of the single-particle wavefunction will remain unchanged despite the change in the reference frame. If it is the function φ_I_(**r**_I_) of the coordinate **r**_I_ in the crystal I frame, it will be the function φ_II_(**r**_II_) = φ_I_(**r**_I_) of the coordinate **r**_II_ = *M* **r**_I_ of the same point in the crystal II frame. Therefore, the relevant transformation of the crystal I (quantum) state |φ_I_(*z*)〉 into the crystal II one, 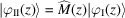 , can be obtained by explicit construction as done by Sasso *et al.* (2022 ▸), but now extended to a non-null roll angle. The result is (see Section 3 in the supporting information) 

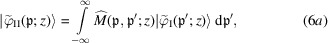

where 

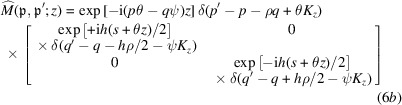

and (*p*′, *q*′) and (*p*, *q*) are the variables conjugate to (*x*_I_, *y*_I_) and (*x*_II_ − *s*, *y*_II_), respectively. Since we assumed *z*_II_ = *z*_I_, we approximated the transformation by rotating the crystal II diffracting planes, not the crystal itself.

The Dirac-delta arguments *p* + ρ*q* − θ*K*_*z*_ and *q* ± *h*ρ/2 + ψ*K*_*z*_ encode the changes of the 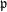 -mode propagation direction seen by crystal II. The ±θ*z* phase terms encode the displace­ment of crystal II along the *x* axis due to the yaw angle θ. The phase factor 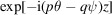 , which is common to the *o* and *h* states, corresponds to geometric optics. It encodes that the incoming rays seen by crystal II are translated by 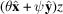 . This phase factor is essential to account for the difference between the free-space propagations from mirrors 1 and 2 to the analyser. The first is carried out in the crystal I reference frame, the second in the crystal II one.

Eventually, the propagation through the interferometer is given by 

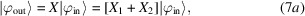

where, by concatenating the crystal and free-space transfer matrices and taking the (possible) misalignment of the split crystals into account, 

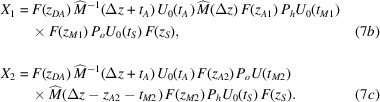


*X*_1_ and *X*_2_ propagate the initial |φ_in_〉 state along the first and second arms of the interferometer, respectively, *P*_*n*_ projects into the |*n*〉 state, and the meaning of the geometric quantities is shown in Figs. 2 ▸, 3 ▸ and 4 ▸.

Since the reciprocal-space representation of *X*_*i*_ is not diagonal, the propagation of 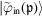 requires an integration. Hence, 

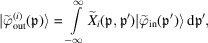

where 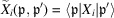 is a 2 × 2 matrix and *i* = 1, 2 labels the interferometer arm. Here and in the following, we omit to indicate the source and detector *z* coordinates, which are implied in the in and out subscripts.

To achieve the maximum visibility of the interference fringes, the interferometer geometry must be free of aberration, that is, θ = ρ = ψ = 0 rad, *t*_*S*_ = *t*_*A*_, *t*_*M*1_ = *t*_*M*2_, *z*_*A*1_ = *z*_*M*2_ and *z*_*A*2_ = *z*_*M*2_, and consequently the defocus Δ*z* = (*z*_*A*1_ − *z*_*M*1_) − (*z*_*M*2_ − *z*_*A*2_) is null.

## 4. Input wave: partially coherent source

To take the partial coherence of the source into account, we describe each incoming particle as in the probabilistic superposition of the (separable) single-particle Gaussian wave packets (Appendix *B* [App appb]), 

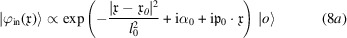

or, by using the reciprocal-space representation, 

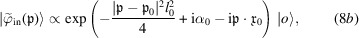

where 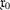 is the origin, *l*_0_ is the radius and 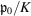 is the propagation angle to the **K**_*o*_ wavevector.

Here, 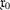 , α_0_ and 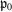 are uncorrelated normal variables having zero mean. Without loss of generality, we made the *x*_0_ mean and reference-frame origins coincide and, for the sake of simplicity, assumed circular profiles for both the single-particle states and their superposition.

After averaging over these single-particle states (see Section 4 in the supporting information), we obtain a Gaussian Schell model of the source (Schell, 1967 ▸; Mandel & Wolf, 1995 ▸; Wolf, 2007 ▸). The relevant 2 × 2 position-space representation of the density matrix is 

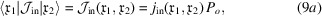

where 

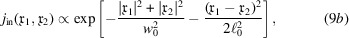

ℓ_0_ ≃ *l*_0_/(1 + *l*_0_σ_0_) measures the coherence length, σ_0_ is the standard deviation of the 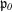 distribution, *w*_0_ ≫ ℓ_0_ is the 1/*e* ^2^ source radius and 

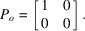

By using the 2 × 2 reciprocal-space representation, we have 

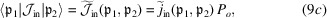

where, provided 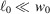 , 

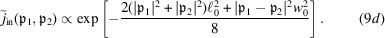




Propagation of the density matrix is given by 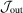 = 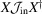 , where the dagger indicates the conjugate transpose (Appendix *B* [App appb]). The diagonal elements 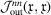 and 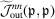 are the particle densities of the *n* = *o*, *h* states in the chosen basis.

The density matrix associated with a completely incoherent superposition is diagonal with identical elements. That associated with a coherent (Gaussian) single-particle state |φ_in_〉 having radius *w*_0_ corresponds to (9*b* [Disp-formula fd9b]), where 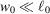 and, consequently, the term proportional to 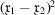 is omitted (see Section 4 in the supporting information). The reciprocal-space representation (9*c* [Disp-formula fd9c]) is accordingly recalculated as 

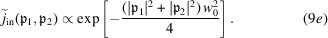




## 5. Exit waves: coherent source

When making explicit the components of (7*a* [Disp-formula fd7a]), (7*b* [Disp-formula fd7b]) and (7*c* [Disp-formula fd7b]) to calculate the |φ_out_〉 components, the resulting algebra is quite abstruse so the reader is referred to Section 5 in the supporting information. Here, therefore, we start by considering the simplest case of a coherent initial state. If the initial single-particle state is 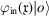 , by application of (7*a* [Disp-formula fd7a])–(7*c* [Disp-formula fd7b]), the final state, after travelling through the interferometer, is 

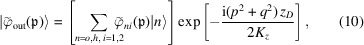

where *z*_*D*_ is the detector distance from the source and (see Section 5 in the supporting information) 

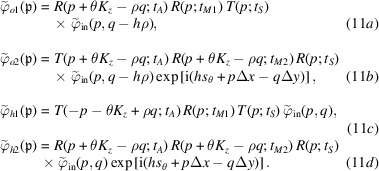

These equations concatenate the relevant reflection and transmission coefficients of the splitter, mirrors and analyser. The θ*K*_*z*_ − ρ*q* offset of the arguments and *hs*_θ_ phase originate from the transformation of the second-crystal chain of transfer matrices to take the misalignment of the split crystals into account. Free-space propagations are responsible for the exponential factor of (10), which encodes diffraction, and the *p*Δ*x* − *q*Δ*y* phase.

**Figure 3:**
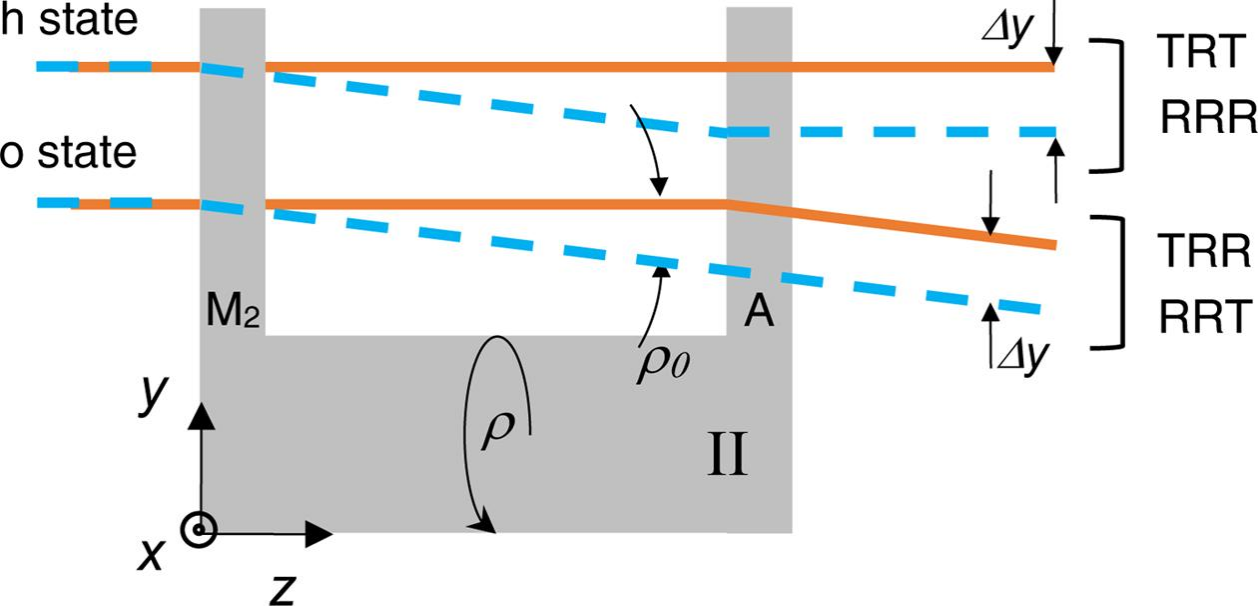
Skew-symmetric triple Laue interferometer with split crystals (crystal II, side view). The rays leaving the interferometer in the *o* and *h* states are shifted vertically. Orange and blue indicate the first and second arms, respectively. Δ*y* = 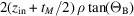 is the vertical offset between the interfering beams. ρ_0_ = 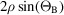 is the (vertical) reflection angle of crystal II. ρ is the mutual pitch angle of the split crystals (the rotation angle about the *z* axis).

We omitted the second-order terms. 

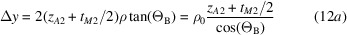

is the vertical offset between the interfering rays leaving the source collinearly or that at the source between those interfering collinearly (Fig. 3 ▸), 

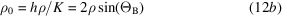

is the vertical component of the angle of reflection of the particle bouncing off the crystal II diffracting planes (Fig. 3 ▸), and 

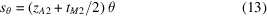

is the mutual displacement (along the *x* axis) between the analyser and mirror 2 when crystal II rotates by an angle θ. The horizontal offset of the interfering plane waves (Fig. 2 ▸), 

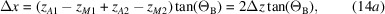

is null when the defocus 

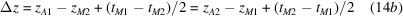

[where *z*_*F*1_ = *z*_*M*2_ + (*t*_*M*2_ − *t*_*M*1_)/2 and *z*_*F*2_ = *z*_*M*1_ + (*t*_*M*1_ − *t*_*M*2_)/2 (Fig. 2 ▸) are the focal plane distances from the mirrors] is zero.

Free-space propagation separates the *o* and *h* states leaving the interferometer into two spatially localized states, 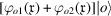 and 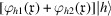 , whose *i* = 1, 2 components interfere. Therefore, in equations (11*a* [Disp-formula fd11])–(11*d*), we omitted non-essential phase terms shared by the *i* = 1, 2 waves and associated the phase difference between the interfering waves with 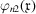 .

If the detector counts the total particles per time unit, the observed signals are 

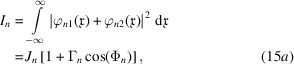

where, using the Parseval theorem to carry out the integrations in reciprocal space, the mean count rate, interference, visibility and phase are given, respectively, by 

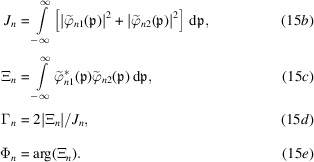

**Figure 4:**
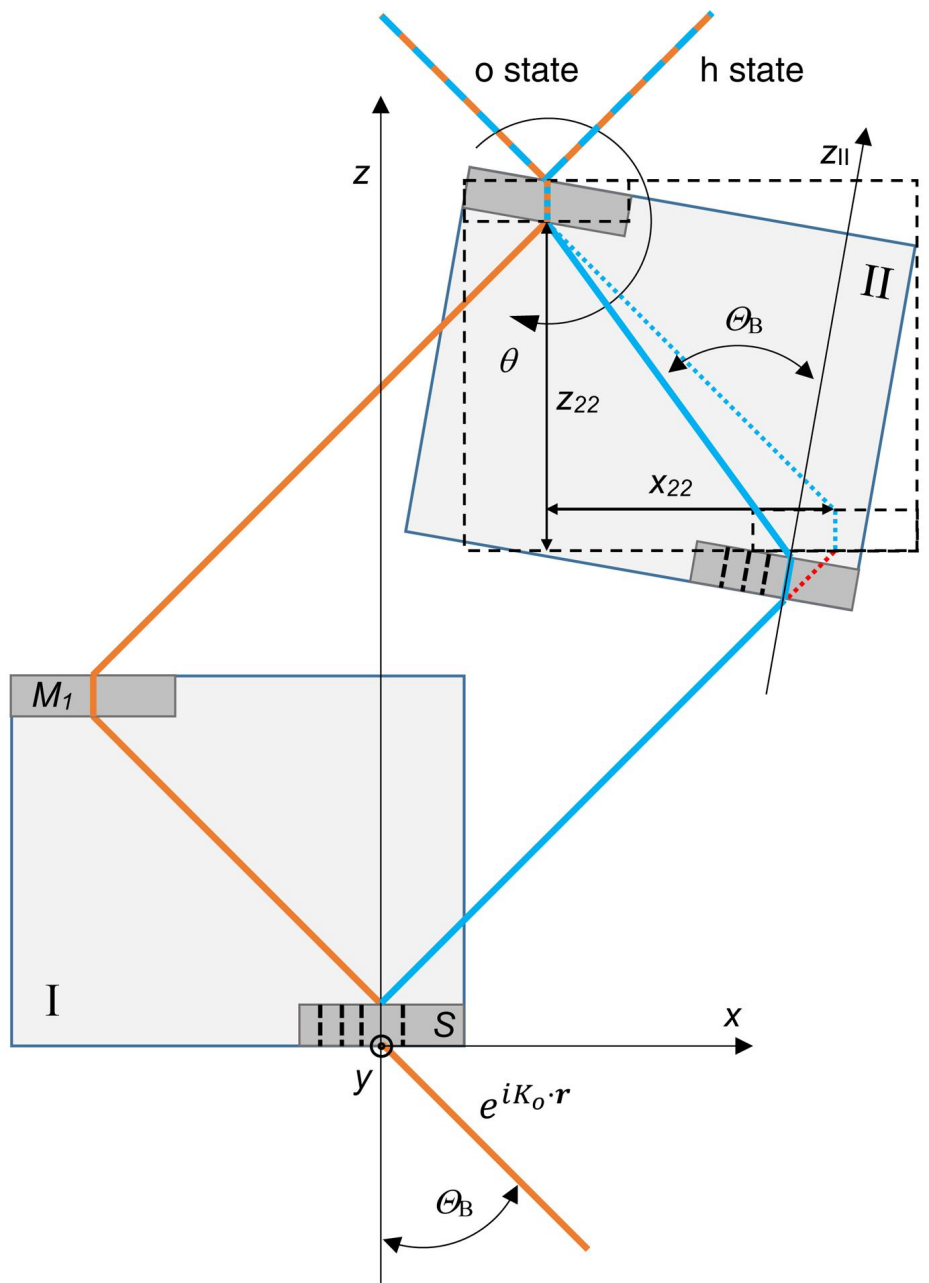
Skew-symmetric triple Laue interferometer with split crystals (top view). θ is the mutual yaw angle of the split crystals (the rotation angle about the *y* axis). For the sake of simplicity, the interferometer geometry is ideal. Orange and blue rays indicate arms 1 and 2, respectively; the dashed lines indicate the second arm of the aligned interferometer. The solid and dotted paths inside crystal II have the same length. The dotted red line is the path difference **Δ**_OPD_. The rays forward transmitted by the two mirrors have been omitted.

The phase *hs*_θ_ in (11*b* [Disp-formula fd11]) and (11*d* [Disp-formula fd11]) is the foundation of angle measurements by crystal X-ray interferometry (Windisch & Becker, 1992 ▸; Kuetgens & Becker, 1998 ▸). A rotation by an angle θ between the split crystals changes the interference phase by *hs*_θ_ = *h*(*z*_*A*2_ + *t*_*M*2_/2)θ. As a result, it yields travelling fringes, the period of which depends on the crystal separation *z*_*A*2_ + *t*_*M*2_/2. By noting that *h* = 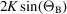 , this phase can be rewritten as 

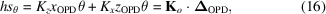

where *z*_OPD_ = *z*_*A*2_ + *t*_*M*2_, 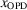 = 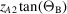 , 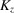 = 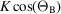 , 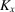 = 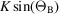 and **Δ**_OPD_ is the length difference of the interferometer arms (Fig. 4 ▸).

Therefore, two equivalent descriptions of the angular sensitivity of the interferometer are possible. Firstly, from the viewpoint of the incoming particles, mirror 2 moves by 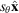 relative to the analyser but the arm lengths are unchanged. Secondly, from the standpoint of crystal II, the source and crystal I are displaced rigidly and the arm lengths change.

As in the symmetric counterpart, a defocused skew-symmetric interferometer moves the interfering waves apart horizontally by 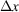 = 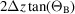 and this shift is encoded – via the ‘time-shifting’ property of the Fourier transform – by the phase difference *p*Δ*x* in (11*b* [Disp-formula fd11]) and (11*d* [Disp-formula fd11]).

Differently from what happens with its symmetric counter­part and as shown in Fig. 4 ▸, the pitch misalignment of the split crystals does not misalign the interfering waves but moves them apart vertically by Δ*y*, and this shift is encoded by the phase difference *q*Δ*y* in (11*b* [Disp-formula fd11]) and (11*d* [Disp-formula fd11]). This raises questions about the effect of source coherence that are answered in the next section.

The interferometer is insensitive to the displacements and roll rotations of the two blocks. In fact, the *s* and ψ degrees of freedom disappear from equations (11*a* [Disp-formula fd11])–(11*d* [Disp-formula fd11]). Also, by neglecting gravity and the Coriolis force, it is insensitive to displacements along the *z* axis. In fact, a constant added to *z*_*M*2_ and *z*_*A*1_ does not change the defocus, which is the only quantity in equations (11*a* [Disp-formula fd11])–(11*d*) that depends on the *z* coordinate. These insensitivities make the skew-symmetric geometry the best choice to obtain long and variable interferometer arms.

Last, but not least, equations (11*a* [Disp-formula fd11])–(11*d*) do not evidence fast differential phase variations in the interfering beams associated with the ρ angle between the split crystals, as reported by Becker & Bonse (1974 ▸).

## 6. Exit waves: partially coherent source

The propagation of the particles emitted by a partially coherent source through the interferometer – a linear system described by the transfer matrix *X* – differs from (7*a* [Disp-formula fd7a]) only because the 2 × 2 density matrix 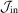 takes the place of the single-particle wavefunction |φ_in_〉 as the propagated quantity. Therefore (Feynman, 2018 ▸; Cohen-Tannoudji *et al.*, 2019 ▸), 

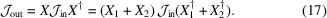

The position- and reciprocal-space densities of the particles leaving the interferometer in the *n* = *o*, *h* states, 

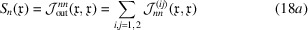

and 

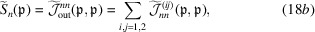

are the diagonal elements of the 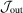 representations 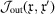 = 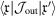 and 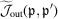 = 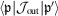 . The elements of these representations are linked by 

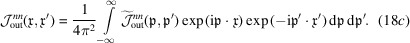

The *ij* elements of the propagated density matrix are 

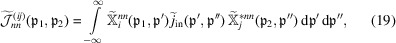

where 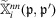 are the *nn* = *oo*, *hh* diagonal elements of the 2 × 2 matrix 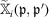 = 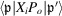 . The operators *X*_*i*_ [equations (7*a* [Disp-formula fd7a])–(7*c* [Disp-formula fd7b])] concatenate the reflection and transmission coefficients of the splitter, mirrors and analyser and propagate the initial mixed state along the *i* = 1, 2 arms. The projector *P*_*o*_ originates from 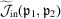 = 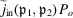 [equation (9*c* [Disp-formula fd9c])]. More details of the derivation of (19) are given by Sasso *et al.* (2022 ▸). The results of integration (19) are given in Appendix *C* [App appc].

The integration of the particle density gives the total counts of the particles leaving the interferometer in the *n* = *o*, *h* states. Hence, the equivalents of equations (15*a* [Disp-formula fd15a])–(15*e* [Disp-formula fd15b]) are 

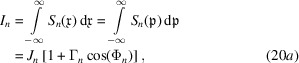

where 

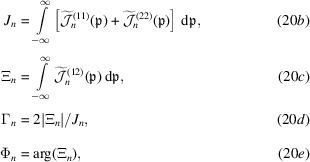

and the 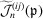 expressions are given in Appendix *C* [App appc].

The matrix elements 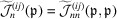 in equations (20*a* [Disp-formula fd20a])–(20*e* [Disp-formula fd20b]) are the incoherent dual of 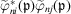 in equations (15*a* [Disp-formula fd15a])–(15*e* [Disp-formula fd15b]). More precisely, by disentangling its algebraic structure, we can see that (19) propagates every single-particle wave packet in the probabilistic superposition that describes the source, calculates every product 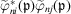 , and averages them over the random origins, phase and propagation direction.

A noteworthy result [equations (34*a* [Disp-formula fd34a])–(34*h*) in Appendix *C* [App appc]] is that, assuming a Gaussian Schell model of the source, equations (20*a* [Disp-formula fd20a])–(20*e* [Disp-formula fd20b]) differ from (15*a* [Disp-formula fd15a])–(15*e* [Disp-formula fd15b]) only by a scale factor. Therefore, they describe the integrated interference observed when the source is Gaussian with a radius equal to the coherence length ℓ_0_. Also, the spherical wave approximation of the initial state (Authier, 2001 ▸) corresponds to ℓ_0_ = 0 and an incoherent source.

### 6.1. Moiré fringes

The pitch misalignment ρ shifts the interfering waves vertically by 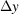 = 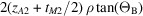 . This offset stems from the *q*Δ*y* phase of 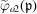 and 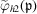 [equations (11*a* [Disp-formula fd11])–(11*d*)] because of the ‘time-shifting’ property of the Fourier transform. Owing to the curvature of the interfering wavefronts, it yields a pattern of horizontal fringes.

The particle density (18*a* [Disp-formula fd18a]) encodes this pattern. To see this, let us consider the *h* state and the *q* factor of 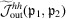 . By neglecting the *q*ρ offset in the argument of the reflection and transmission coefficients, the *p* factor of 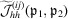 is irrelevant. Hence, the terms of interest in (18*b* [Disp-formula fd18b]) [see also equations (33*a* [Disp-formula fd33a])–(33*i* [Disp-formula fd33b])] are 

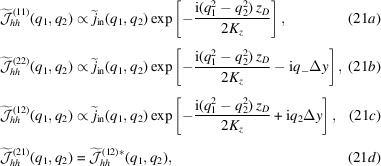

where 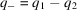 , 

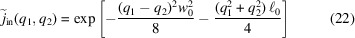

and *z*_*D*_ is the detector distance from the source.

The result of the inverse Fourier transform (18*c* [Disp-formula fd18c]) is (see Section 6.1 in the supporting information) 

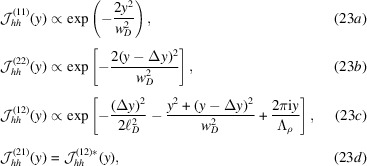

where ℓ_*D*_ and *w*_*D*_ are, respectively, the correlation length and beam size at the detector plane *z* = *z*_*D*_ (Appendix *B* [App appb]). Finally, the position-space density of the particles leaving the interferometer in the *h* state (18*a* [Disp-formula fd18a]) is 

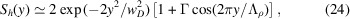

where 

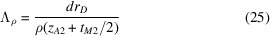

is the fringe period, 1/*r*_*D*_ is the principal curvature of the particle density 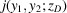 at the detector plane [equation (32*c* [Disp-formula fd32a]) in Appendix *B* [App appb]] and 

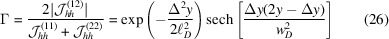

is the interference visibility.

The fringe period Λ_ρ_ is the same as that of the moiré pattern originating from the superposition of two (cylindrical) wavefronts having the same radius of curvature 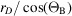 and propagating parallel at the Θ_B_ angle to the *z* axis and at the Δ*y* vertical distance.

Regarding the visibility Γ, the hyperbolic secant comes from the different amplitudes of the interfering beams at the vertical coordinate *y* [equations (23*a* [Disp-formula fd23]) and (23*b* [Disp-formula fd23])]. It expresses the visibility of the fringes generated by a coherent source and corresponds to the ℓ_*D*_ → ∞ limit of (26). In this case, the visibility is a maximum (actually, one) when *y* = Δ*y*/2, because the interfering beams have equal amplitude. It is also a maximum when *w*_*D*_ → ∞, because in such a case the wavefront curvature is null. The exponential factor, where Δ*y* = 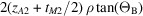 and ρ is the pitch angle between the split crystals, takes the partial coherence of the source into account.

To give a numerical example, with a divergent beam originating from a point source 2 m away from the detector, a 0.1 m mirror-to-analyser separation and (220) reflecting planes, the fringe period is Λ_ρ_ = 3.8 mm µrad/ρ.

## 7. Results

The interferometer operation has been numerically simulated to study its sensitivity to geometric aberrations and to quantify the machining and alignment tolerances to attain a satisfactory functioning. The values of the parameters used in the simulations are given in Table 1 ▸. Owing to the significant X-ray absorption, the choice of 15.7Λ_*e*_ ≃ 0.619 mm for the crystal thickness follows the objective of combined X-ray and neutron interferometry.

### 7.1. Crystal thicknesses

If the interferometer geometry is ideal, that is, *t*_*S*_ = *t*_*A*_, *t*_*M*1_ = *t*_*M*2_, Δ*z* = 0 and θ = ρ = 0 rad, the visibility of the interference of the particles that leave the interferometer in the *o* state is one. Therefore, the thickness of the interferometer crystals can be optimized by maximizing the visibility of the interference of the particles that leave the interferometer in the *h* state. As anticipated in the *Introduction* [Sec sec1], two cases are worth considering, (i) *t*_*M*1_ = *t*_*M*2_ = *t*_*S*_ = *t*_*A*_ and (ii) *t*_*M*1_ = *t*_*M*2_ = 2*t*_*S*_ = 2*t*_*A*_ (Bauspiess *et al.*, 1976 ▸; Becker *et al.*, 2001 ▸).

**Figure 5:**
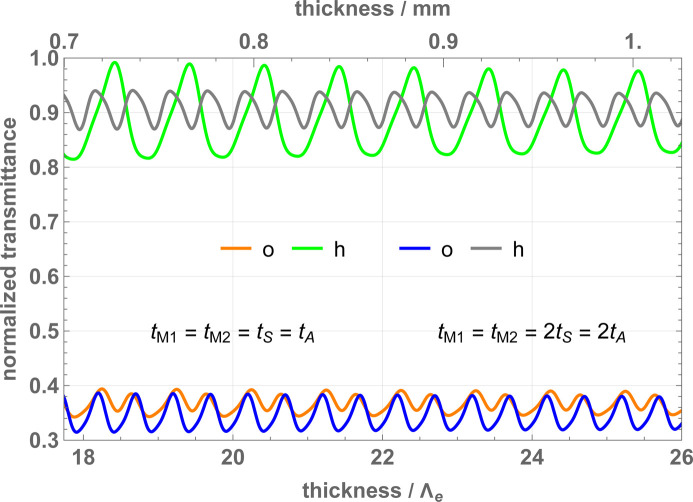
Transmission coefficients of a perfectly aligned interferometer versus the crystal thicknesses. The orange and green lines refer to the output *o* and *h* states, respectively, when the crystals have the same thickness. The blue and grey lines refer to the output *o* and *h* states, respectively, when the mirror crystals have double thickness. The transmissions in the *h* output state (top lines) are greater than those in the *o* one (bottom lines) because of the greater number of individual forward transmissions contributing to them. The parameters used in the calculations are given in Table 1 ▸.

Fig. 5 ▸ shows the transmittances of the interferometer from the initial to the final, *n* = *o*, *h*, states. They are calculated as 

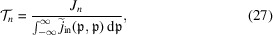

where the particle density 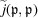 is given by (9*d* [Disp-formula fd9d]) and the correlation length ℓ_0_ is set to 10 nm.

Due to the interferometer’s limited angular acceptance, the transmittance depends on the angular width of the initial state. The larger the former, the smaller the latter. The oscillations, having periods of Λ_*e*_ and Λ_*e*_/2, are what remains of the pendellösung fringes. They originate in the periodicity of the transmission and reflection coefficients (4*b* [Disp-formula fd4b]) and (4*c* [Disp-formula fd4b]) and are damped by the scattering of the initial *p* modes. It is worth noting that the crystal thickness is the dual of the laser pulse duration in atom interferometry.

**Figure 6:**
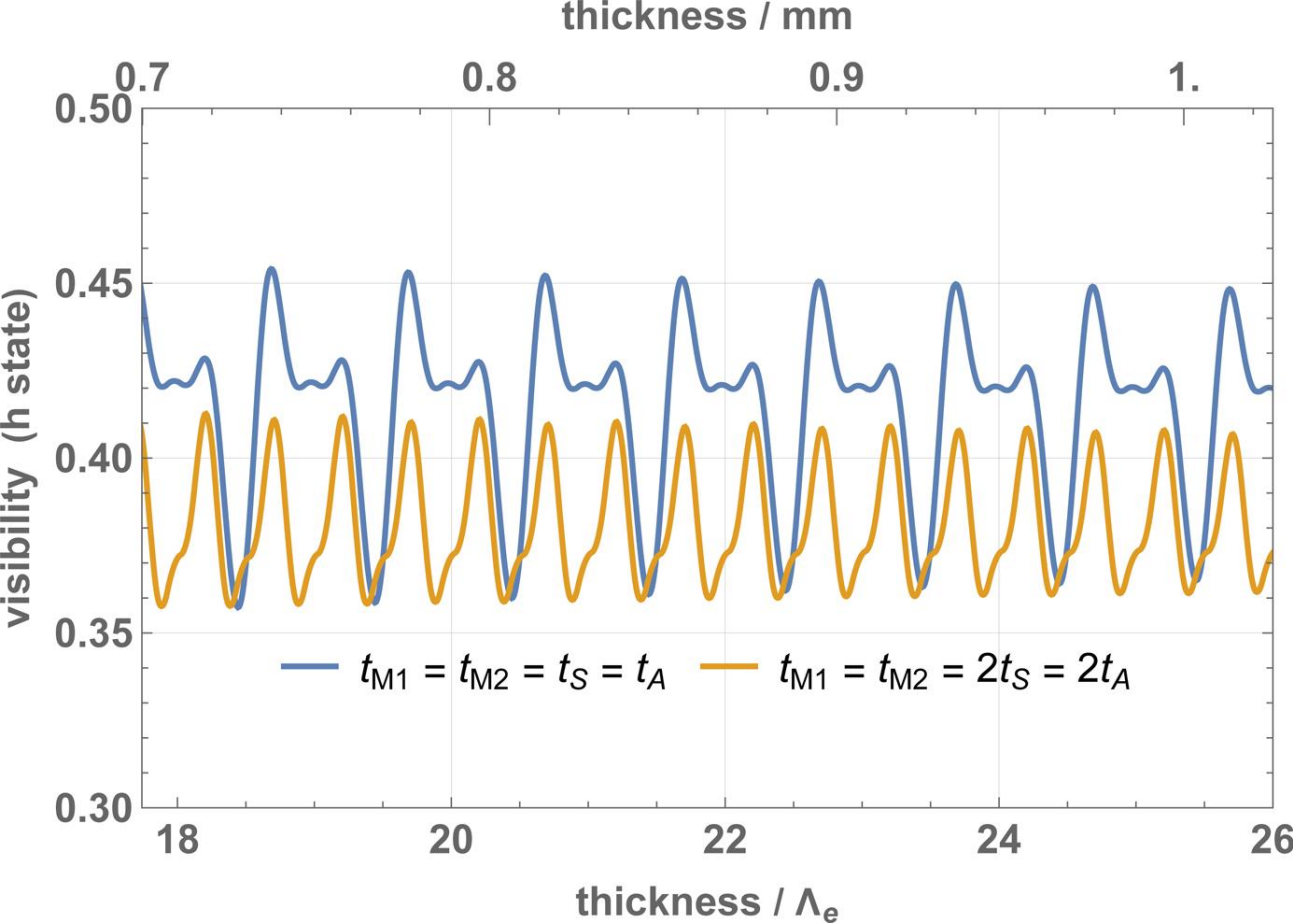
Interference fringe visibilities (*h* state) of a perfectly aligned interferometer versus the crystal thicknesses. The parameters used in the calculations are given in Table 1 ▸.

The visibility of the *h*-state fringes is given in Fig. 6 ▸. Pendellösung fringes are again visible, having periodicity Λ_*e*_/2 [case (ii)] and Λ_*e*_ [case (i)]. Both the particle densities and visibility are maxima when *t*_*S*_ = *t*_*A*_ ≃ (*m* + 0.7)Λ_*e*_, where *m* is an integer. This condition will be adopted from now on.

### 7.2. Rocking curves

**Figure 7:**
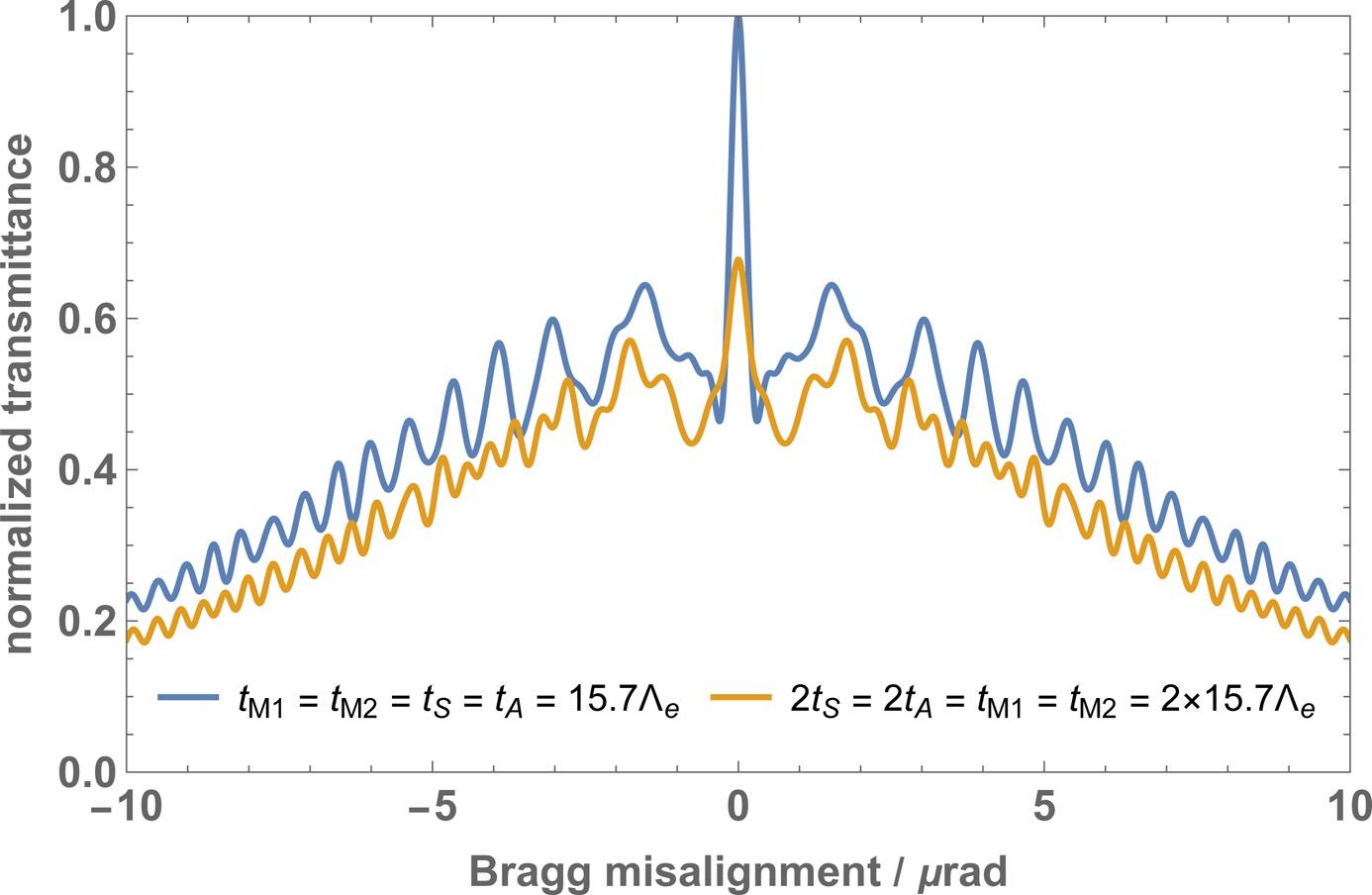
Triple reflection rocking curves 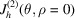 versus the mutual yaw angle of the split crystals θ. The parameters used in the calculations are given in Table 1 ▸. The mutual pitch angle ρ is null.

The interferometer rocking curves are given by the total counts 

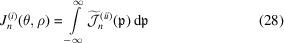

of the particles leaving the interferometer in the *n* = *o*, *h* states and crossing the interferometer along the *i* = 1, 2 arms. Among them, the triple reflection rocking curve 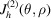 plays a significant role in the alignment of the split-crystals’ yaw angles. This is shown in Fig. 7 ▸ where the pitch angle ρ between the split crystals is null.

The ρ*q* offset of the arguments of the *R*(*p* + θ*K*_*z*_ − ρ*q*; *t*_*A*_) and *R*(*p* + θ*K*_*z*_ − ρ*q*; *t*_*M*2_) factors of 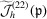 [equation (34*f* [Disp-formula fd34a])] shifts the exact Bragg alignment of the *q* mode from θ = 0 to θ = ρ*q*/*K*_*z*_. Therefore, if the mutual pitch angle of the split crystals is not null, the integration (28) of 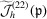 over *q* washes out the pendellösung fringes. In particular, it washes out the central peak of Fig. 7 ▸. This peak loss might be used to align the pitch angles of the split crystals.

**Figure 8:**
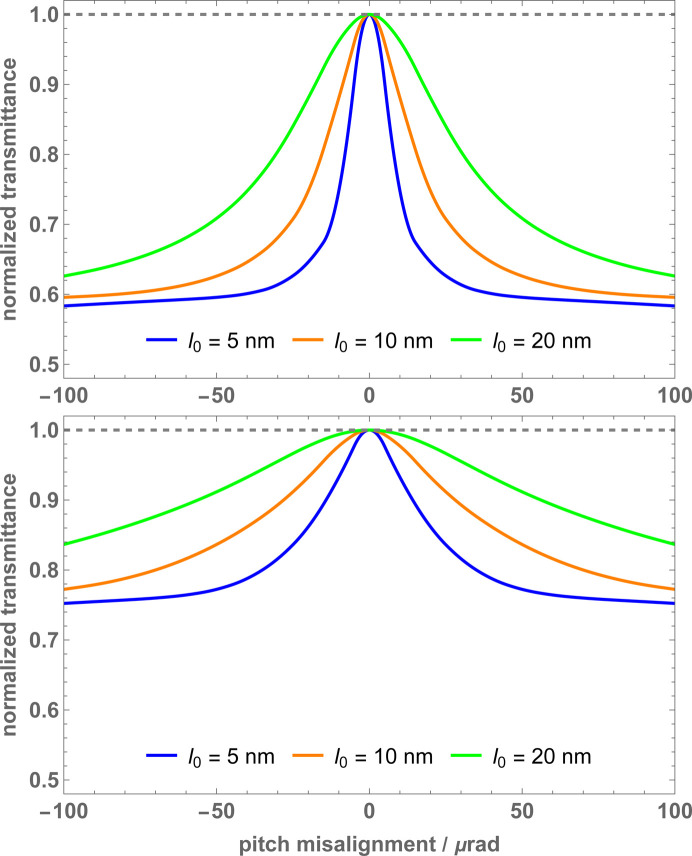
Rocking curves 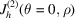 versus the mutual pitch angle of the split crystals ρ. The transmittances are normalized so that they have unit maxima. (Top) *t*_*M*1_ = *t*_*M*2_ = *t*_*S*_ = *t*_*A*_ = 15.7Λ_*e*_ ≃ 0.619 mm. (Bottom) *t*_*M*1_ = *t*_*M*2_ = 2*t*_*S*_ = 2*t*_*A*_ = 2 × 15.7Λ_*e*_ ≃ 1.239 mm. The parameters used in the calculations are given in Table 1 ▸. ℓ_0_ is the coherence length. The mutual yaw angle θ is null. The horizontal dashed line pertains to coherent plane-wave illumination.

Fig. 8 ▸ shows how the height of the θ = 0 peak depends on the mutual pitch angle ρ. The rocking curve generated by a Gaussian Schell model of the source is the same as that yielded by a fully coherent Gaussian source having a radius equal to the coherence length ℓ_0_ (see Appendix *C* [App appc]). Therefore, the greater the coherence, the greater the collimation of the initial state and the greater the interferometer transmittance. For this reason, in Fig. 8 ▸, to show how the peak visibility depends on the source coherence, we normalized all maxima to the same (unit) value.

In the case of coherent plane-wave illumination (*i.e.* if *K*ℓ_0_ → ∞ and *Kw*_0_ → ∞), *q* = 0 and the rocking curves are insensitive to the pitch misalignment. As the source coherence decreases, the central peak of the triple-reflection rocking curve disappears. In fact, the smaller coherence length and collimation imply a greater spread of the *q* modes.

### 7.3. Fringe visibility

**Figure 9:**
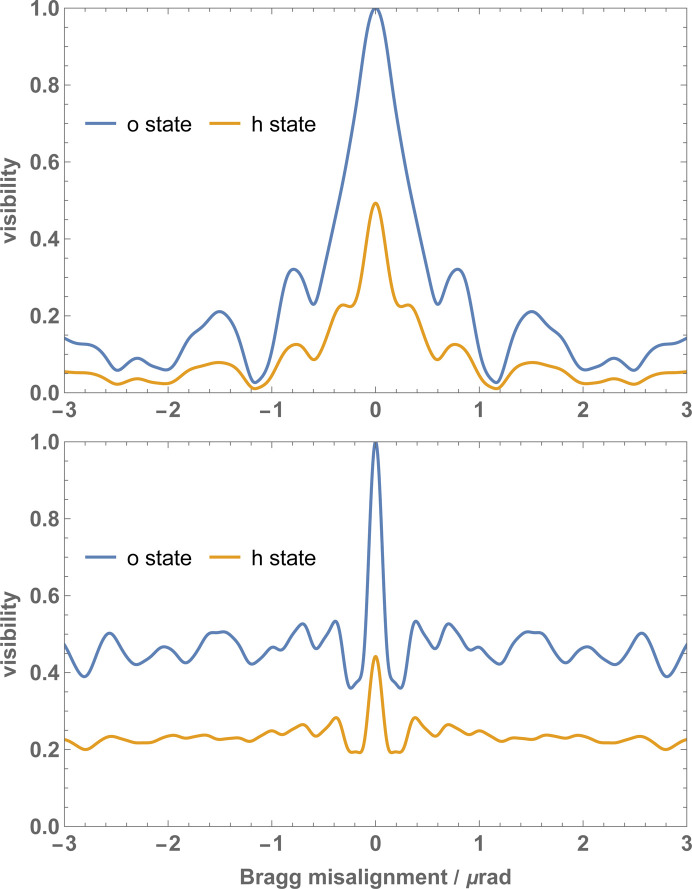
Fringe visibility versus the mutual yaw angle θ of the split crystals. The parameters used in the calculations are given in Table 1 ▸. (Top) *t*_*M*1_ = *t*_*M*2_ = *t*_*S*_ = *t*_*A*_ = 15.7Λ_*e*_ ≃ 0.619 mm. (Bottom) *t*_*M*1_ = *t*_*M*2_ = 2*t*_*S*_ = 2*t*_*A*_ = 2 × 15.7Λ_*e*_ ≃ 1.239 mm. The mutual pitch angle ρ is null.

As shown in Fig. 9 ▸, the yaw misalignment causes a loss of interference visibility, which is due to the averaging of the pendellösung fringes implied by (20*c* [Disp-formula fd20b]). If *z*_*M*1_ = *z*_*M*2_ = 2*z*_*S*_ = 2*z*_*A*_, the interference also preserves a significant contrast when θ ≠ 0. In fact, if Ξ_*n*_ is approximated by averaging the oscillating terms of 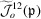 and θ ≠ 0, the result is zero. However, if *z*_*M*1_ = *z*_*M*2_ = 2*z*_*S*_ = 2*z*_*A*_, some of the arguments of the trigonometric functions that replace the products and powers of sines and cosines in 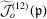 [equations (34*c* [Disp-formula fd34a]), (4*b* [Disp-formula fd4b]) and (4*c* [Disp-formula fd4b])] are null and thus the function value is independent of the misalignment. Consequently, in this case and only in this case, averaging the oscillating terms of 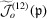 does not nullify Ξ_*n*_. The price to pay is the strictest alignment required to achieve the maximum visibility.

**Figure 10:**
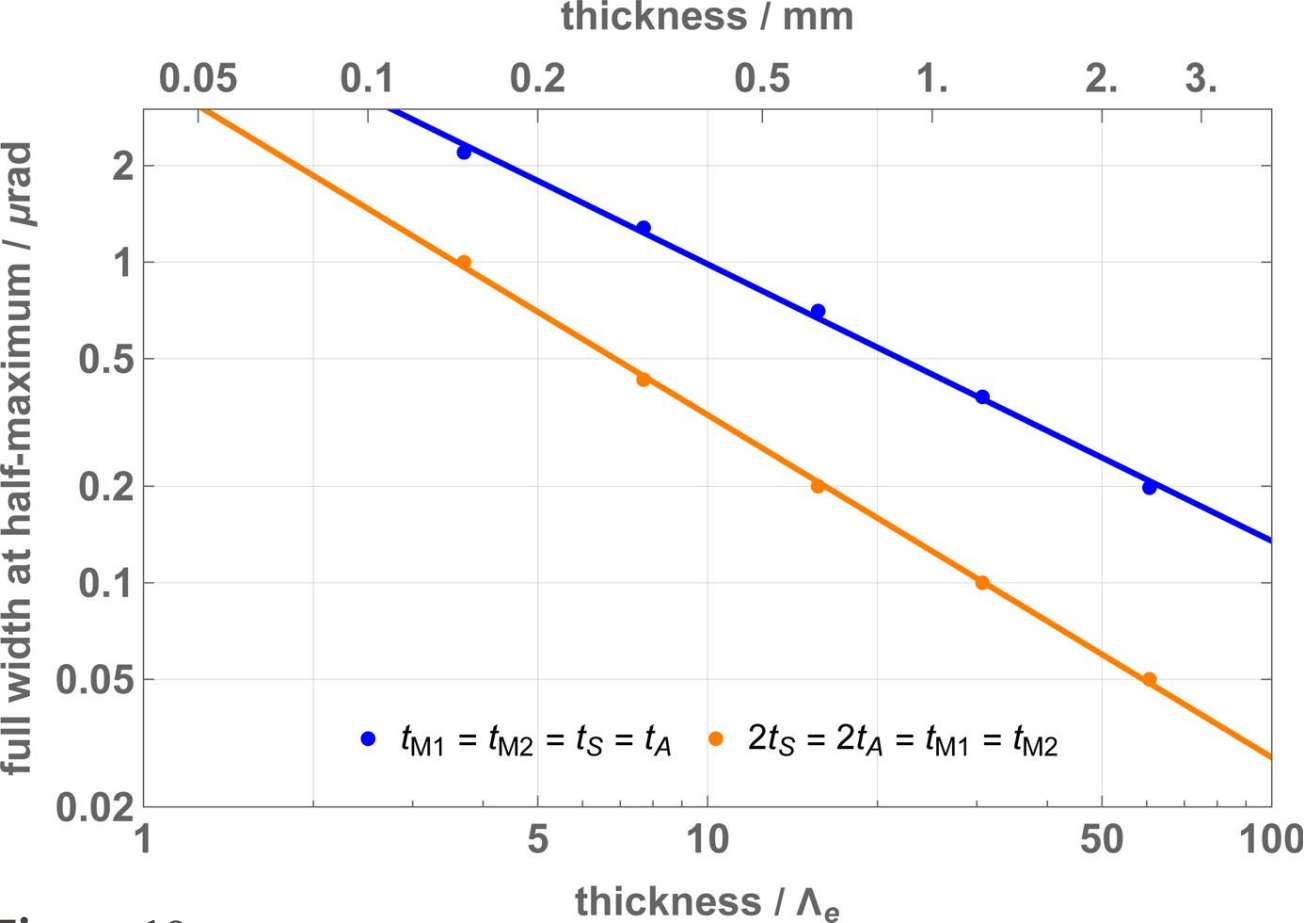
The full width at half-maximum of the θ = 0 visibility peak (*o* state, see Fig. 9). The parameters used in the calculations are given in Table 1 ▸.

The sensitivity of the visibility to the yaw misalignment depends on the crystal thickness. Fig. 10 ▸ shows the full width at half-maximum of the θ = 0 peak for the interference of the particles that leave the interferometer in the *o* state. The thinner the crystals, the better.

The loss of visibility due to the pitch misalignment can be investigated by considering the *q* factor of 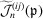 [equations (34*a* [Disp-formula fd34a])–(34*h* [Disp-formula fd34a])] and neglecting the *q*ρ offset in the argument of the reflection and transmission coefficients so that the *p* factor of 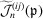 is irrelevant. Also, for the sake of simplicity, we limit the analysis to the *h* state. By application of equations (20*a* [Disp-formula fd20a])–(20*d* [Disp-formula fd20b]), we obtain (see Section 7.3 in the supporting information) 

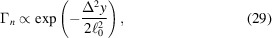

where 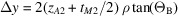 is the vertical offset of the interfering rays.

With a skew-symmetric splitting of the crystals, the pitch misalignment is less critical than with the symmetric splitting used to demonstrate the feasibility of the interferometer alignment. In fact, unlike what happens when only the analyser is separated and the interfering beams are mutually tilted by the analyser’s reflection, the loss of visibility depends only on the coherence length ℓ_0_. As examined in Appendix *D* [App appd] and the supporting information, the rationale of the visibility being independent of the beam size and detector distance is the parallelism of the interfering beams.

**Figure 11:**
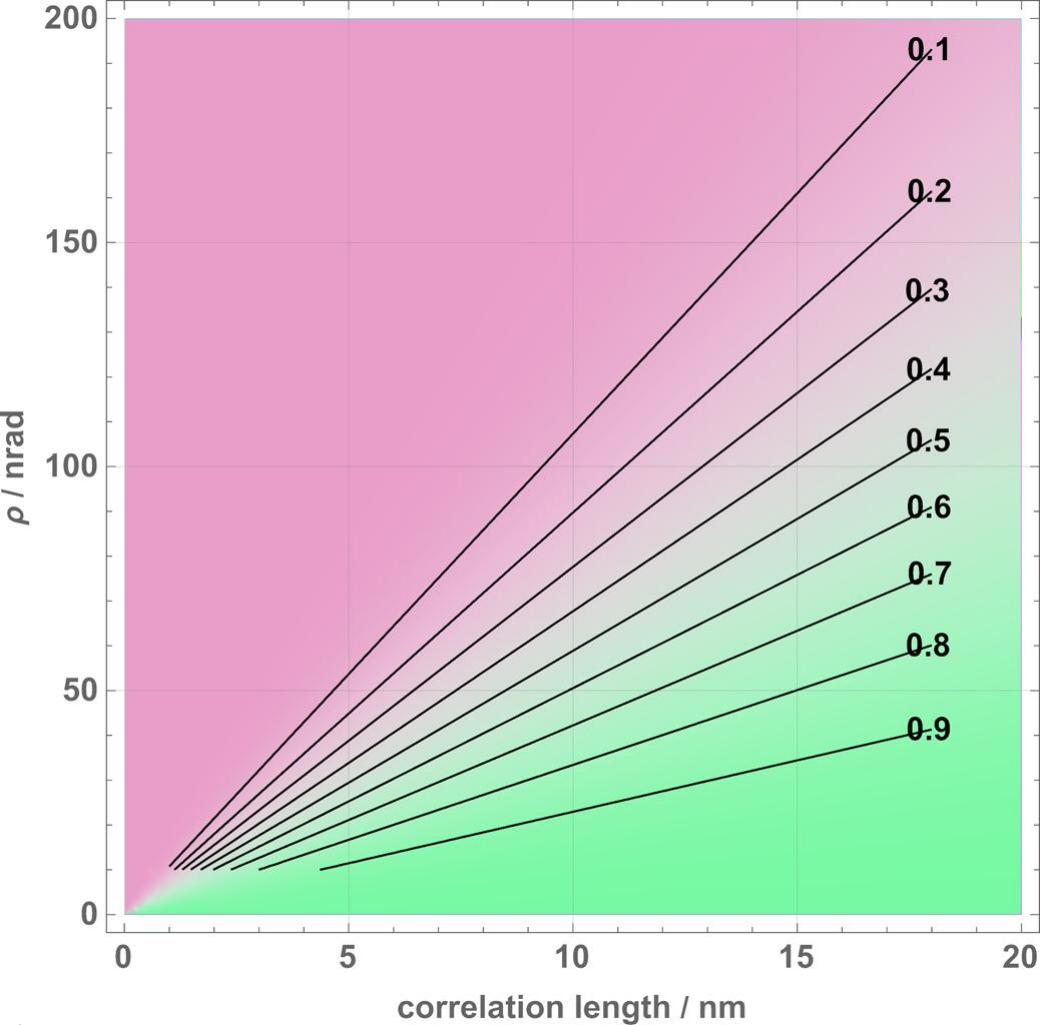
An example of the visibility Γ_*o*_(ℓ_0_, ρ) versus the correlation length ℓ_0_ and mutual pitch angle ρ (ideal geometry and *o* state) [equation (29)]. Purple denotes Γ_*o*_ = 0 and green Γ_*o*_ = 1. The black lines are contours corresponding to the indicated visibility levels. The analyser-to-mirror distance *z*_*A*2_ + *t*_*M*2_/2 is 0.1 m. The Bragg angle is 45°.

If the illumination is coherent (*i.e.* if 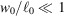 ) then equation (29), where *w*_0_ substitutes for ℓ_0_, holds. This is because equations (20*a* [Disp-formula fd20a])–(20*e* [Disp-formula fd20b]) describe equivalently the operation of an interferometer illuminated by a fully coherent Gaussian beam having a source size equal to ℓ_0_. When both *Kw*_0_ and *K*ℓ_0_ tend to infinity, the illumination is a plane wave and the visibility is insensitive to the pitch misalignment. This is consistent with the insensitivity of a skew-symmetric interferometer to the mutual slide of the split crystals and the null curvature of the interfering wavefronts. A nomogram showing (29) as a function of the correlation length ℓ_0_ and pitch angle ρ is given in Fig. 11 ▸.

## 8. Monte Carlo simulation

We used Monte Carlo simulations to evaluate how manufacturing errors affect the visibility of the interference fringes and the uncertainty of the measurement of their phase.

### 8.1. Fringe visibility

To maximize the interferometer transmittivity and the visibility of the *h*-state interference, the thickness of the interferometer crystals was set to (i) *t*_*M*1_ = *t*_*M*2_ = *t*_*S*_ = *t*_*A*_ = 15.7Λ_*e*_ ≃ 0.619 mm and (ii) *t*_*M*1_ = *t*_*M*2_ = 2*t*_*S*_ = 2*t*_*A*_ = 2 × 15.7Λ_*e*_ ≃ 1.239 mm.

Although the splitter–analyser and mirror pairs should have the same thickness and a null defocus, the removal of surface damage after cutting causes geometric imperfections. To simulate them, the actual thicknesses were obtained by adding random errors sampled from a zero mean uniform distribution having [−*u*, *u*] support, *u* being the targeted manufacturing tolerance. In addition, a zero mean defocus Δ*z* was sampled from the same uniform distribution. The crystals were assumed to be perfectly aligned and therefore the yaw and pitch angles were always set to zero. There were 10 000 simulation runs.

**Figure 12:**
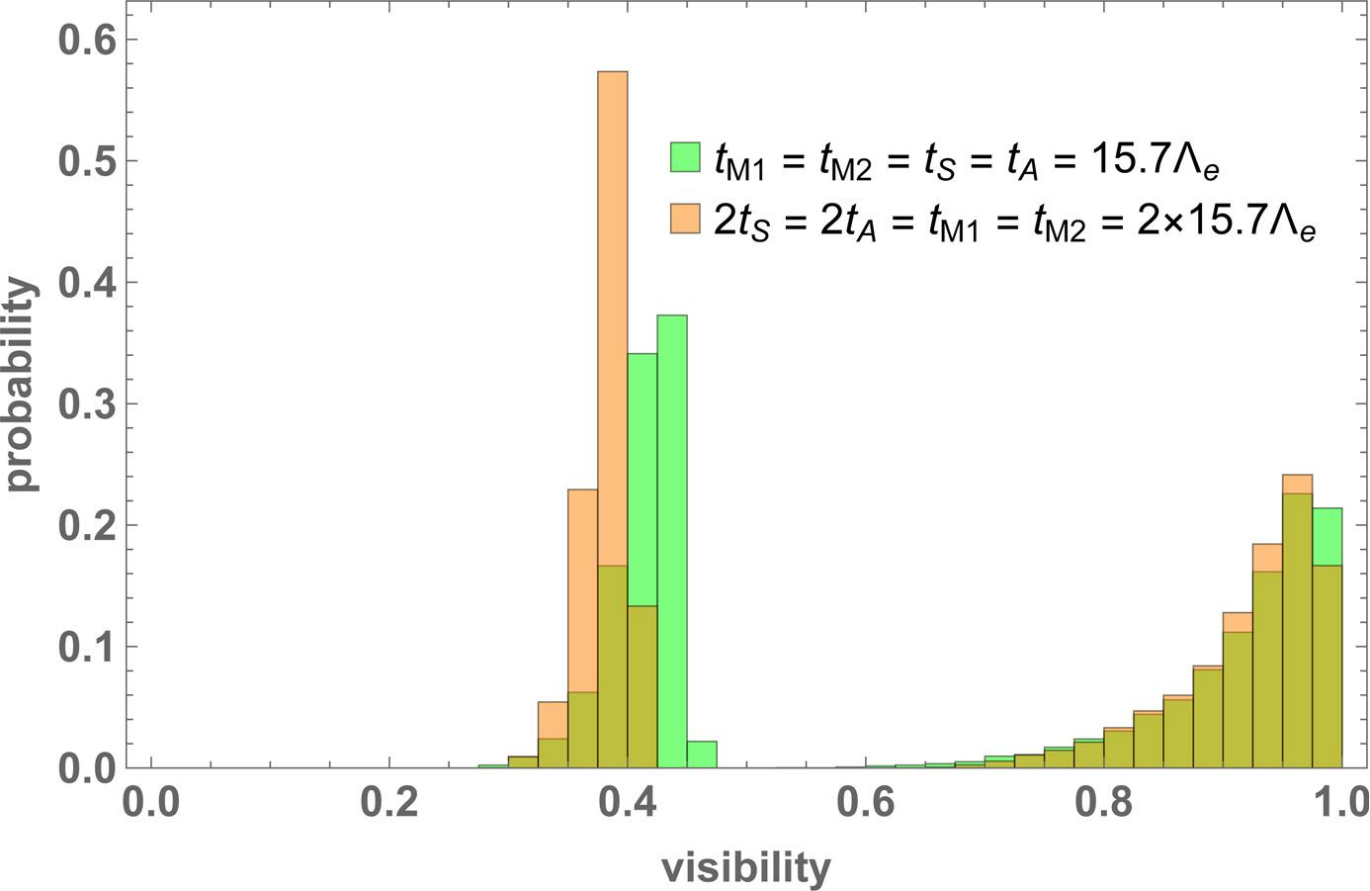
A histogram of the interference visibilities, (right) *o* state and (left) *h* state. The interferometer is perfectly aligned. The manufacturing tolerance is *u* = 2 µm. The parameters used in the Monte Carlo simulations are given in Table 1 ▸.

A visibility histogram when the manufacturing tolerance is *u* = 2 µm and the interferometer is perfectly aligned, that is, the crystals’ mutual yaw and pitch angles are null, is shown in Fig. 12 ▸. The differences between cases (i) and (ii) are tiny but visible. On average, case (i), *i.e.* all the crystals having the same thickness, ensures a slightly greater visibility, mainly when the post-selected state is the *h* one.

### 8.2. Phase uncertainty

In the absence of extraneous noise, the utmost accuracy of the fringe-phase determination is set by the dual wave and particle nature of X-rays and neutrons, which produces a count noise. The standard uncertainty of the phase estimate 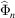 is given by (Bergamin *et al.*, 1991 ▸) 

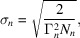

where *n* = *o*, *h* and *N*_*n*_ is the number of particles counted. Accordingly, for any given observation duration and source brilliance, the accuracy depends only on the fringe visibility and transmission coefficient.

Assuming that both the *o* and *h* signals are considered and that the 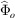 and 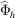 estimates are weighted, the measured phase is 

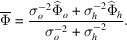

The standard uncertainty of 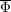 is 

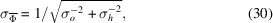

where, factoring the common proportionality factor 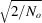 , the standard uncertainties of 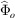 and 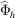 are normalized as 

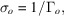




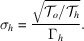

The transmittances 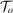 and 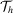 are given by (27) and 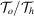 = 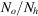 .

**Figure 13:**
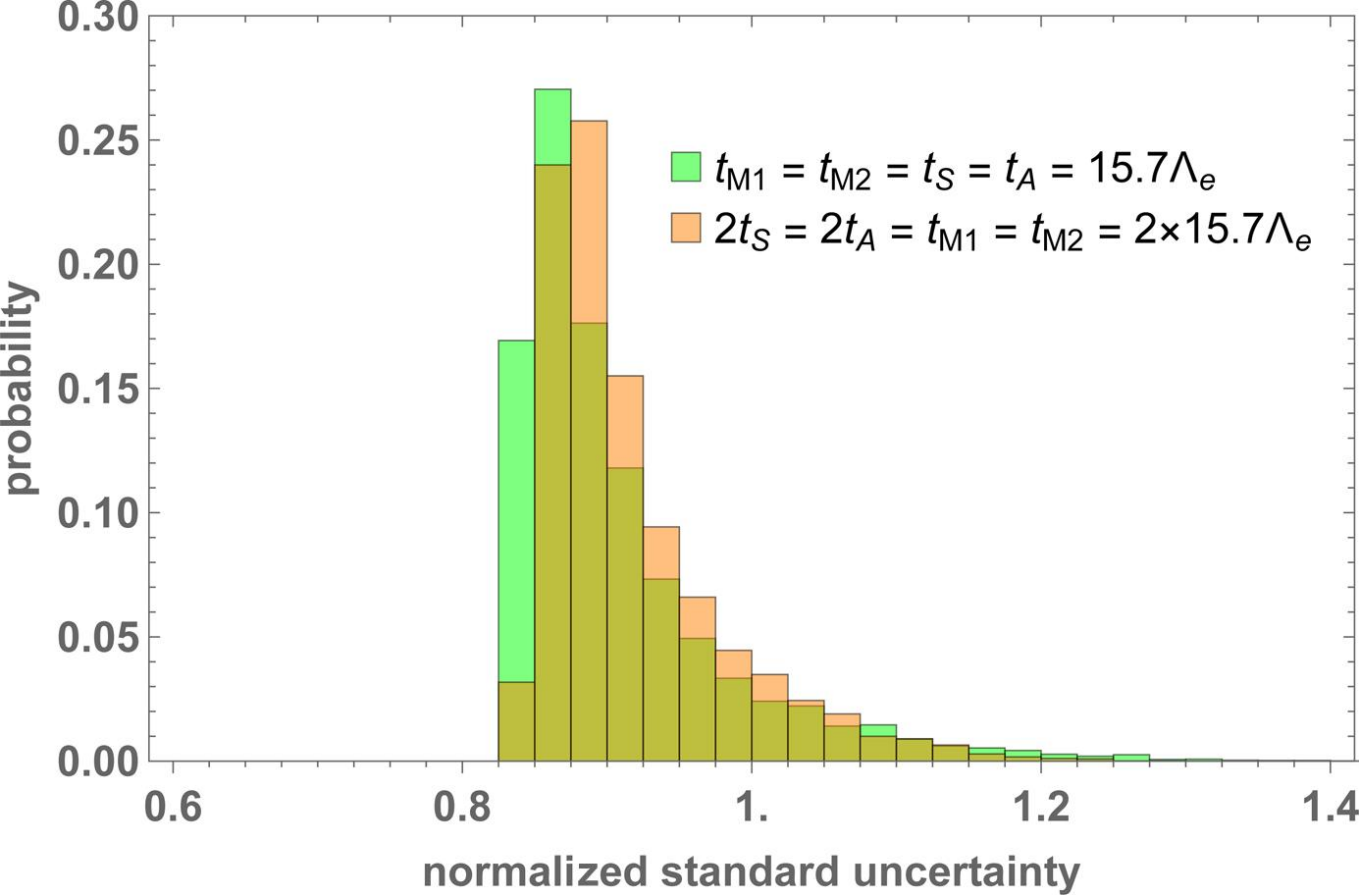
A histogram of the phase estimate uncertainties [equation (30)]. The manufacturing tolerance is *u* = 2 µm. The parameters used in the Monte Carlo simulations are given in Table 1 ▸.

Fig. 13 ▸ shows the 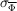 histograms for the (i) and (ii) geometries (defined in Section 8.1). The manufacturing tolerances were set to *u* = 2 µm and we assumed that the interferometer was perfectly aligned. Because of the normalization adopted, the uncertainty associated with the 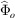 estimate is always greater than one. The unit value is achieved when the interferometer geometry is ideal and the mutual yaw and pitch angles of the split crystals are null. The smallest uncertainty in Fig. 13 ▸ is less than one because we considered the weighted mean of the 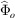 and 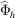 estimates. Crystals having the same thickness still ensure slightly better accuracy.

**Figure 14:**
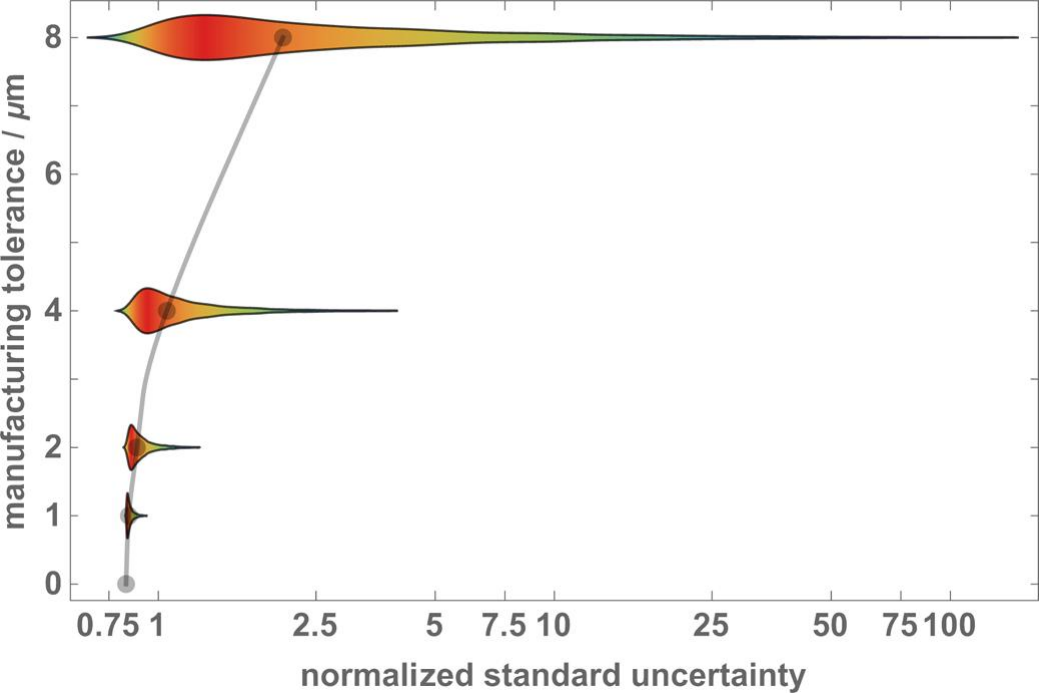
Violin plots of the phase estimate uncertainties [equation (30)]. The manufacturing tolerances are 0, 1, 2, 4 and 8 µm. The parameters used in the Monte Carlo simulations are given in Table 1 ▸. The crystal thickness is *t*_*M*1_ = *t*_*M*2_ = *t*_*S*_ = *t*_*A*_ = 15.7Λ_*e*_ ≃ 0.619 mm. Dots are the data medians. The *u* = 2 µm plot is from the histogram in Fig. 13.

To investigate how the manufacturing errors affect the accuracy of the phase estimate, we repeated the Monte Carlo simulation with manufacturing errors sampled from uniform distributions having *u* = ±(0, 1, 2, 4, 8) µm supports. The results are recapped as violin plots in Fig. 14 ▸.

## 9. Discussion

For a triple Laue split-crystal interferometer, two geometries are possible, symmetric and skew-symmetric. In the symmetric one, the two mirrors are at equal distances from the splitter, that is, *z*_*M*1_ = *z*_*M*2_ (Fig. 2 ▸). In this case, the crystal splitting means the analyser is free to move. However, since the distance of the analyser from the mirrors is constrained to be the same as the splitter distance, there is no freedom to change the length of the interferometer arms. Such interferometers allowed measuring the lattice parameter of ^28^Si (Massa *et al.*, 2011 ▸, 2020 ▸) and led to the determination of the Avogadro constant (Fujii *et al.*, 2018 ▸) and the realization of the kilogram by counting atoms (Massa *et al.*, 2020 ▸).

To increase the arms’ separation and length the skew-symmetric geometry is necessary. In this case, the interferometer consists of two independent crystal blocks, as shown in Fig. 2 ▸. Since it is insensitive to parallel translation of one block with respect to the other, they can be placed far apart, so long arms are possible and their length can also be varied in real time. This opens the way to experiments testing fundamental symmetries and interactions, such as the relation between quantum mechanics and gravity.

To align and operate the interferometer, two angles (the mutual yaw and pitch rotations, about the *y* vertical axis and *z* optical axis, respectively) must be zero. Also, the residual mechanical vibrations and the long measurement times imply that an optical interferometer must be hosted in the gap between the crystals to monitor these angles in real time and actively nullify them with, typically, sub-nano­radian accuracy. In particular, the interference phase is sensitive to the crystals’ Bragg alignment. The farther apart the arms, the greater the sensitivity. Luckily, the sensitivity of both crystal and optical interferometers increases equally with arm separation. Nanoradian metrology and technologies have already been developed in the measurement of the Si lattice parameter (Ferroglio *et al.*, 2008 ▸; Massa *et al.*, 2009 ▸), in lattice comparators (Mendenhall *et al.*, 2023 ▸) and in γ-ray spectroscopy (Krempel, 2011 ▸; Massa *et al.*, 2013 ▸). However, an order of magnitude improvement is necessary.

To achieve a reasonable fringe visibility also far from the perfect Bragg alignment, Windisch & Becker (1992 ▸) proposed mirror crystals with twice the thickness of the splitter and analyser. However, the maximum visibility peak is sharper than that occurring with the equal-thickness choice. We have observed that, in both cases, the angular width of the visibility peak increases as the crystal thickness decreases.

For the first time we have quantified from first principles the effects of the source coherence and pitch misalignment of the split crystals on the visibility and phase of the interference fringes. We found that, apart from a scale factor and using a Gaussian Schell model of the source, the reciprocal-space densities of the particles leaving the interferometer are the same as those yielded by a fully coherent initial Gaussian state having a radius equal to the coherence length of the source. Unless one is interested in the spatial pattern of the interference, this significantly simplifies the analysis.

Varying the mutual pitch of the split crystals, Becker & Bonse (1974 ▸) observed unexplained travelling fringes in the integrated intensities of the beams leaving the interferometer. We did not find clues to this potentially troublesome effect. It may have been caused by an undetected parasitic change in the Bragg alignment accompanying the pitch misalignment.

The goal of interferometry is to determine the phase difference between the split beams. The minimum uncertainty depends on the interference visibility, in addition to the particle counts. The higher, the better. Therefore, we examined how the manufacturing tolerance impacts the visibility and uncertainty of the phase estimate. A 3 µm tolerance seems to be the maximum permitted before a significant loss of accuracy is observed. We also found that this tolerance is mostly insensitive to the crystal thicknesses.

## 10. Conclusions

Adapting the mathematical framework described in a previous paper (Sasso *et al.*, 2022 ▸), we have modelled a split-crystal skew-symmetric interferometer and investigated its sensitivity to crystal thicknesses, misalignments and machining tolerances. Taking the source coherence and three-dimensional operation into account was necessary to quantify, from first principles, the effect of the pitch misalignment on the pattern and visibility of the interference fringes. We found that a partially coherent source is equivalent to a coherent one having a radius equal to the coherence length. This result simplified the numerical implementation of the model.

We did not consider gravity, the Coriolis force, or residual acceleration due to seismic and environmental noise. They will be the subject of future work.

Owing to the extreme sensitivity of the interference phase, operating such an interferometer requires an order of magnitude improvement in the metrology and control capabilities of the mutual yaw angle of the split crystals.

## Supplementary Material

Mathematica notebook. DOI: 10.1107/S1600576723010245/ei5097sup1.txt

PDF of rendered notebook script. DOI: 10.1107/S1600576723010245/ei5097sup2.pdf

## Figures and Tables

**Table 1:** Parameters used in the numerical simulations The thickness *t*_*S*_ = *t*_*A*_ ≃ (*m* + 0.7)Λ_*e*_ maximizes the interferometer transmission and fringe visibility. The choice of 15.7Λ_*e*_ ≃ 0.619 mm is consequential to the objective of combined X-ray and neutron interferometry.

χ_0_ = χ_*h*_ = −2.382 × 10^−6^	ν = −1
*n*_0_ = 1–1.191 × 10^−6^	μ_0_ = 0
λ = 0.272 nm	*d* = 192 pm
*K* = 23.1 rad nm^−1^	*h* = 32.7 rad nm^−1^
Λ_*e*_ = 39.4 µm	Θ_B_ = 0.786 rad = 45°
Δ*z* = 0	ℓ_0_ = 5, 10, 20 nm
Case (i): *t*_*M*1_ = *t*_*M*2_ = *t*_*S*_ = *t*_*A*_ = 15.7Λ_*e*_	Case (ii): *t*_*M*1_ = *t*_*M*2_ = 2*t*_*S*_ = 2*t*_*A*_ = 2 × 15.7Λ_*e*_

## Appendix A Representations of the single-particle state

The position- and reciprocal-space representations of the single-particle state |φ_*n*_(*z*)〉 are the superpositions

and

The orthogonality and completeness of the  =   and  =  bases are expressed by the integral representations of the delta distribution

and

## Appendix B Free-space propagation

Propagating the Gaussian wave packet,

where *l*_0_ is the initial (*z* = 0) radius measured in the *xy* plane (assumed the same along the *x* and *y* axes), we obtain

where  . The radius *l*_*z*_(*z*) and wavefront curvature 1/*r*_*z*_(*z*) depend on propagation according to

where

is the divergence.

The free-space propagation of the density matrix is given by  =  (Feynman, 2018 ▸; Cohen-Tannoudji *et al.*, 2019 ▸). To give an example, let us consider the *q* factor of  =  [equation (9*d* [Disp-formula fd9d])]. Hence,

After transforming it back to position space, we obtain

where

are the radius, spatial coherence and radius of curvature at a distance *z*, respectively.

The incoherent beam spreads like a wave packet having an initial radius equal to the correlation length ℓ_0_: its divergence is (31*c* [Disp-formula fd31c]), where ℓ_0_ substitutes for *l*_0_, and its spread is dictated by the coherence length ℓ_0_, not by the beam size *w*_0_.

The coherence length increases like the radius of a Gaussian beam having

divergence. Therefore, propagation increases coherence, though negligibly in our case. This is the content of the van Cittert–Zernike theorem.

## Appendix C Propagation of the density matrix

The elements  of the propagated density matrix are given by the integral (19). Using the initial mixed state (9*c* [Disp-formula fd9c]) and writing the results of the integration as

we obtain (see Section 6 in the supporting information)

where *p*_−_ = *p*_1_ − *p*_2_ and *q*_−_ = *q*_1_ − *q*_2_.

By setting  =  and  =  and observing that *p*_−_ = *q*_−_ = 0, the reciprocal-space densities and quantum superpostions of the particles leaving the interferometer in the *n* = *o*, *h* state after crossing the interferometer along the *i*, *j* = 1, 2 arm or both [equation (18*b* [Disp-formula fd18b])] are (see the corresponding section in the supporting information)

where, for a Gaussian Schell model of the source,

By comparing equations (11*a* [Disp-formula fd11])–(11*d*) and (34*a* [Disp-formula fd34a])–(34*h*) we note that, apart from a scale factor, the reciprocal-space densities and quantum superpositions of the particles leaving an interferometer illuminated by the mixed state (9*a* [Disp-formula fd9a])–(9*d* [Disp-formula fd9d]) are the same as those of the particles initially in a fully coherent Gaussian state having a radius equal to the coherence length ℓ_0_. The same is true for the total particle counts, as evident by comparing equations (15*a*)[Disp-formula fd15a]–(15*e* [Disp-formula fd15b]) and (20*a* [Disp-formula fd20a])–(20*e* [Disp-formula fd20b]).

## Appendix D Shearing interferometry

As shown by (29), the visibility of the (integrated) interference fringes depends only on the coherence length ℓ_0_. It is independent of the radius of the interfering beams and detector distance. The reason is that the pitch misalignment of the split crystals separates the interfering beams in the vertical plane but leaves them parallel. To validate this fact, we investigate the interference of two parallel Gaussian wave packets,

spaced by Δ*y*. The integrations over *y* of  ,  and  yield the mean count rates

and quantum superposition

Using (31*a* [Disp-formula fd31a])–(31*c* [Disp-formula fd31c]) to express *l*_*z*_, *r*_*z*_ and  (see Appendix *D* in the supporting information), we obtain the visibility of the integrated interference pattern,

which is independent of the detector distance *z*.

In contrast, if interference occurs between the beams,

which are mutually tilted by an angle ρ_0_ and intersect at *y* = 0, the integration over *y* of  yields (see Appendix *D* in the supporting information) their interference and visibility as

and

which depend on the beam size *l*_*z*_ and thus on the detector distance *z*.

## Appendix E List of the main symbols

: position vector.

: normal to the crystal surface (optical axis).

: **r** component orthogonal to

: reciprocal vector (Fig. 1 ▸).

*d*: diffracting plane spacing (Fig. 1 ▸).

**K**_*o*_, **K**_*h*_ = **K**_*o*_ + **h**: kinematic wavevectors [equation (1*a* [Disp-formula fd1a])].

Θ_B_: Bragg angle (Fig. 1 ▸).

: Bragg law.

: *z* component of **K**_*o*,*h*_ [equation (2)].

: direction cosine.

: variable conjugate to  [equation (3*b* [Disp-formula fd3b])].

*p*: resonance error [equation (3*b* [Disp-formula fd3b])].

ℓ_0_: coherence length [equation (9*b* [Disp-formula fd9b])].

χ_0,*h*_: for X-rays, the Fourier components of the periodic electric susceptibility.

υ_0,*h*_ = −*K* ^2^χ_0,*h*_: for neutrons, the Fourier components of the periodic Fermi pseudo-potential.

*n*_0_ = 1 + Re(χ_0_)/2: refractive index.

μ_0_ = Im(χ_0_)*K*: absorption coefficient.

ν = χ_*h*_/|χ_*h*_|: χ_*h*_ phasor [equations (4*a* [Disp-formula fd4a])–(4*c* [Disp-formula fd4b])].

Λ_*e*_ = 2πγ/(*K*|χ_*h*_|): pendellösung length [equations (4*a* [Disp-formula fd4a])–(4*c* [Disp-formula fd4b])].

: dimensionless resonance error [equations (4*a* [Disp-formula fd4a])–(4*c* [Disp-formula fd4b])].

ζ = π*z*/Λ_*e*_: dimensionless propagation distance [equations (4*a* [Disp-formula fd4a])–(4*c* [Disp-formula fd4b])].

*t*_*S*_, *t*_*M*1_, *t*_*M*2_, *t*_*A*_: crystal thicknesses.

Δ*z*: defocus [equation (14*b* [Disp-formula fd14b])].

*z*_*A*_, *z*_*D*_: analyser and detector distances from the source.

Δ*y*: separation of the interfering rays [equation (12*a* [Disp-formula fd12a])].

Δ*x*: separation of the interfering rays [equation (14*a* [Disp-formula fd14a])].

θ: rotation angle about  (yaw) [equation (5*b* [Disp-formula fd5b])].

ρ: rotation angle about  (pitch) [equation (5*b* [Disp-formula fd5b])].

ψ: rotation angle about  (roll) [equation (5*b* [Disp-formula fd5b])].

|φ_in_〉, |φ_out_〉: pure-state initial and final wavevectors [equations (8*a* [Disp-formula fd8a]), (8*b* [Disp-formula fd8b]), (10)].

: mixed-state initial and final density matrices [equations (9*a* [Disp-formula fd9a])–(9*d* [Disp-formula fd9d]), (17)].

*F*(*z*), *U*_0_(*z*), *X*: transfer matrices (free-space, crystal, interferometer, respectively) [equations (3*c* [Disp-formula fd3c]), (4*a* [Disp-formula fd4a])–(4*c* [Disp-formula fd4b]), (7*a* [Disp-formula fd7a])–(7*c* [Disp-formula fd7b])].

[−*u*, +*u*]: support of the manufacturing errors.

*n* = *o*, *h*: state components (label).

*i* = 1, 2: interferometer arm (label).
