# Supplementary material for: Operation model of a skew-symmetric split-crystal neutron interferometer
Source: J Appl Crystallogr. 2024 Feb 1;57(Pt 1):44–59. doi: 10.1107/S1600576723010245 (PMC10840307; doi:10.1107/S1600576723010245)
Supplement: Supplementary file 2 [file j-57-00044-sup2.pdf]

JOURNAL OF APPLIED CRYSTALLOGRAPHY

Volume 57 (2024)

Supporting information for article:

Operation model of a skew-symmetric split-crystal neutron interferometer

Carlo P. Sasso, Giovanni Mana and Enrico Massa

Operation model of a skew-symmetric split-crystal neutron interferometer

Carlo Paolo Sasso [1], Giovanni Mana [1,2], and Enrico Massa [1]

1) INRIM – Istituto Nazionale di Ricerca Metrologica, Torino, Italy
2) UNITO – Università di Torino, Dipartimento di Fisica, Torino, Italy

3. Interferometer operation

focus coordinates, Eq. (5c)

```
Clear["Global`*"]; Remove["Global`*"];

e1 = zM1 - zF1 == - zM2 + zF2;
(* same x-axis displacement of the rays along the two paths *)
e2 = zM1 + tM1 + zF1 == zM2 + tM2 + zF2; (* same distance from the splitter *)
zF12 = Solve[{e1, e2}, {zF1, zF2}][[1]];
xF == Simplify[(zM1 - zF1) Tan[ΘB] /. zF12] (* x coordinate *)
xF == Simplify[(-zM2 + zF2) Tan[ΘB] /. zF12] (* x coordinate *)
zF == Simplify[tS + zM1 + tM1 + zF1 /. zF12] (* z coordinate *)
```

$$xF == \frac{1}{2}\,(tM1 - tM2 + 2\,zM1 - 2\,zM2)\,\mathrm{Tan}[\Theta B]$$

$$xF == \frac{1}{2}\,(tM1 - tM2 + 2\,zM1 - 2\,zM2)\,\mathrm{Tan}[\Theta B]$$

$$zF == \frac{tM1}{2} + \frac{tM2}{2} + tS + zM1 + zM2$$

rotation matrix

```
Clear["Global`*"]; Remove["Global`*"];

R = {{1, -ρ, θ}, {ρ, 1, -ψ}, {-θ, ψ, 1}};
Print["R = ", R // MatrixForm]
(* inverse of the rotation matrix, first order *)
Ri = {{1, ρ, -θ}, {-ρ, 1, ψ}, {θ, -ψ, 1}};
```

$$R = \begin{pmatrix} 1 & -\rho & \theta \\ \rho & 1 & -\psi \\ -\theta & \psi & 1 \end{pmatrix}$$

transformation $\hat{M}(z)$: restriction to V_2

```
rII = {xII, yII, zII};
(* Ko and Kh unit vectors; 0 ==> o, 1 ==> h *)
K[n_] = K {If[n == 0, -1, 1] × Sin[θB], 0, Cos[θB]};
arg = Expand[K[#].(Ri.(rII - {s, 0, 0}) - rII) & /@ {0, 1}] /.
    {Cos[θB] → Kz / K, Sin[θB] → h / (2 K)} /. xII → xII + s;
arg = Simplify[arg];
Print["⟨o|M̂|o⟩ = Exp[ⅈ Ko.(M^-1rII - rII)] = ", Exp[I arg[[1]]]]
Print["⟨h|M̂|h⟩ = Exp[ⅈ Kh.(M^-1rII - rII)] = ", Exp[I arg[[2]]]]
```

⟨o|M̂|o⟩ = Exp[ⅈ Ko.(M^-1rII - rII)] = $e^{i\left(\frac{1}{2} h\,(s+zII\,\theta-yII\,\rho)+Kz\,(xII\,\theta-yII\,\psi)\right)}$

⟨h|M̂|h⟩ = Exp[ⅈ Kh.(M^-1rII - rII)] = $e^{i\left(-\frac{1}{2} h\,(s+zII\,\theta-yII\,\rho)+Kz\,(xII\,\theta-yII\,\psi)\right)}$

transformation $\hat{M}(z)$: restriction to $\mathcal{L}_2$

```
rI = {xI, yI, zI};
dd = Apply[DiracDelta, Ri.(rII - {s, 0, 0}) - rI] /. xII → xII + s /.
   -zI + zII + xII θ - yII ψ → -zI + zII;
Print["⟨rII|M̂|rI⟩ = ", dd]
```

⟨rII|M̂|rI⟩ = DiracDelta[-zI + zII, xI - xII + zII θ - yII ρ, -yI + yII - xII ρ + zII ψ]

transformation $\hat{M}(z)$: direct space representation

```
M = DiagonalMatrix[Exp[I arg]] dd;
(DiagonalMatrix[Exp[I arg]] // MatrixForm) dd
```

DiracDelta[-zI + zII, xI - xII + zII θ - yII ρ, -yI + yII - xII ρ + zII ψ]

$$\begin{pmatrix} e^{i\left(\frac{1}{2} h\,(s+zII\,\theta-yII\,\rho)+Kz\,(xII\,\theta-yII\,\psi)\right)} & 0 \\ 0 & e^{i\left(-\frac{1}{2} h\,(s+zII\,\theta-yII\,\rho)+Kz\,(xII\,\theta-yII\,\psi)\right)} \end{pmatrix}$$

transformation $\hat{M}(z)$: reciprocal space representation, Eq. (6b)

```
(* matrix element 11 *)
step11 =
  FullSimplify[InverseFourierTransform[M[[1, 1]], xI, p, FourierParameters → {0, -1}]];
step11 =
  FullSimplify[InverseFourierTransform[step11, yI, q, FourierParameters → {0, -1}]];
step11 = FullSimplify[FourierTransform[step11, xII, p1, FourierParameters → {0, -1}]];
step11 = FullSimplify[FourierTransform[step11, yII, q1, FourierParameters → {0, -1}]];
step11 = step11 / 2 /. -2 p ρ → 0

(* matrix element 22 *)
step22 =
  FullSimplify[InverseFourierTransform[M[[2, 2]], xI, p, FourierParameters → {0, -1}]];
step22 =
  FullSimplify[InverseFourierTransform[step22, yI, q, FourierParameters → {0, -1}]];
step22 = FullSimplify[FourierTransform[step22, xII, p1, FourierParameters → {0, -1}]];
step22 = FullSimplify[FourierTransform[step22, yII, q1, FourierParameters → {0, -1}]];
step22 = step22 / 2 /. 2 p ρ → 0
```

$e^{\frac{1}{2} i (h s + h zII \theta - 2 p zII \theta + 2 q zII \psi)}$ DiracDelta[zI − zII] × DiracDelta[p − p1 + Kz θ − q ρ] × DiracDelta[−2 q + 2 q1 + h ρ + 2 Kz ψ]

$e^{-\frac{1}{2} i (h s + h zII \theta + 2 p zII \theta - 2 q zII \psi)}$ DiracDelta[zI − zII] × DiracDelta[p − p1 + Kz θ − q ρ] × DiracDelta[2 q − 2 q1 + h ρ − 2 Kz ψ]

```
M11 = Simplify[e^(-1/2 i (-2 p z θ+2 q z ψ)) step11 /. DiracDelta[zI - zII] → 1 /. zII → z] /
  DiracDelta[p - p1 + Kz θ - q ρ]
M22 = Simplify[e^(-1/2 i (-2 p z θ+2 q z ψ)) step22 /. DiracDelta[zI - zII] → 1 /. zII → z] /
  DiracDelta[p - p1 + Kz θ - q ρ]
Print[
 "M̂(p,p1;z) = ", (DiagonalMatrix[{M11, M22}] // TraditionalForm) ×
  DiracDelta[p - p1 + Kz θ - q ρ] × Simplify[e^(-1/2 i (-2 p z θ+2 q z ψ))]
]
```

$e^{\frac{1}{2} i h (s + z \theta)}$ DiracDelta[−2 q + 2 q1 + h ρ + 2 Kz ψ]

$e^{-\frac{1}{2} i h (s + z \theta)}$ DiracDelta[2 q − 2 q1 + h ρ − 2 Kz ψ]

M̂(p,p1;z) = $e^{i z (p \theta - q \psi)}$ DiracDelta[p − p1 + Kz θ − q ρ]

$$\begin{pmatrix} e^{\frac{1}{2} i h (s+\theta z)} \delta(-2 q + 2 q1 + h \rho + 2 Kz \psi) & 0 \\ 0 & e^{-\frac{1}{2} i h (s+\theta z)} \delta(2 q - 2 q1 + h \rho - 2 Kz \psi) \end{pmatrix}$$

some tests

definitions

```
Clear["Global`*"]; Remove["Global`*"];

(* free-space propagation *)
F[p_, q_, z_] = {
    {ⅇ^(+ⅈ p z Tan[ΘB]), 0},
    {0, ⅇ^(-ⅈ p z Tan[ΘB])}} ⅇ^(-(ⅈ (p^2+q^2) z)/(2 Kz));
(* transformation M̂(z) *)
Mz1[p_, p1_, q_, q1_, z_] = {
  {ⅇ^((ⅈ h (s+θ z))/2) ⅇ^(-ⅈ (p1 z θ-q1 z ψ)) DiracDelta[p1 - p - ρ q + θ Kz, q1 - q - (h ρ)/2 - ψ Kz], 0},
  {0, ⅇ^(-(ⅈ h (s+θ z))/2) ⅇ^(-ⅈ (p1 z θ-q1 z ψ)) DiracDelta[p1 - p - ρ q + θ Kz, q1 - q + (h ρ)/2 - ψ Kz]}
 };
(* inverse transformation M̂^-1(z) *)
Mz2[p_, p1_, q_, q1_, z_] = Mz1[p, p1, q, q1, z] /. {s → -s, θ → -θ, ρ → -ρ, ψ → -ψ};
```

$\hat{M}^{-1}(z)\,\hat{M}(z)|\psi(z)\rangle = |\psi(z)\rangle$

```
Mz2[p, p1, q, q1, z].Mz1[p1, p2, q1, q2, z].{ψ̃in[p2, q2], 0};
Integrate[%, {p2, -Infinity, +Infinity},
  Assumptions → p1 > 0 && ρ > 0 && q1 > 0 && θ > 0 && Kz > 0];
Integrate[%, {q2, -Infinity, +Infinity},
  Assumptions → p1 > 0 && ρ > 0 && q1 > 0 && θ > 0 && Kz > 0 && h > 0 && ψ > 0];
Integrate[%, {p1, -Infinity, +Infinity},
  Assumptions → ρ > 0 && θ > 0 && Kz > 0 && p > 0 && q > 0];
tmp = Integrate[%, {q1, -Infinity, +Infinity},
   Assumptions → ρ > 0 && θ > 0 && Kz > 0 && h > 0 && ψ > 0 && q > 0];
ExpandAll[%] /. {ρ^2 → 0, θ^2 → 0, ψ^2 → 0, ρ ψ → 0, θ ρ → 0}

{ψ̃in[p, q], 0}
```

$\hat{M}^{-1}(z)\,\hat{F}(z)\,M(0)|\psi(0)\rangle = \mathrm{F}(z)|\psi(0)\rangle$

```
Mz2[p, p1, q, q1, z].F[p1, q1, z].Mz1[p1, p2, q1, q2, 0].{ψ̃in[p2, q2], 0};
Integrate[%, {p2, -Infinity, +Infinity},
  Assumptions → p1 > 0 && ρ > 0 && q1 > 0 && θ > 0 && Kz > 0];
Integrate[%, {q2, -Infinity, +Infinity},
  Assumptions → p1 > 0 && ρ > 0 && q1 > 0 && θ > 0 && Kz > 0 && h > 0 && ψ > 0];
Integrate[%, {p1, -Infinity, +Infinity},
  Assumptions → ρ > 0 && θ > 0 && Kz > 0 && p > 0 && q > 0];
tmp = Integrate[%, {q1, -Infinity, +Infinity},
   Assumptions → ρ > 0 && θ > 0 && Kz > 0 && h > 0 && ψ > 0 && q > 0];
ExpandAll[tmp /. {ρ^2 → 0}] /.
 {ρ^2 → 0, θ^2 → 0, ψ^2 → 0, ρ ψ → 0, θ ρ → 0, h → 2 Kz Tan[ΘB], p q → 0}

{ⅇ^(-(ⅈ p^2 z)/(2 Kz) - (ⅈ q^2 z)/(2 Kz) + ⅈ p z Tan[ΘB]) ψ̃in[p, q], 0}
```

$\hat{M}(z)|\psi(0)\rangle = |\psi(z)\rangle$

```
Mz1[p, p2, q, q2, z].{ψ̃in[p2, q2, z], 0};
Integrate[%, {p2, -Infinity, +Infinity},
  Assumptions → p > 0 && ρ > 0 && q > 0 && θ > 0 && Kz > 0];
Integrate[%, {q2, -Infinity, +Infinity},
  Assumptions → p > 0 && ρ > 0 && q > 0 && θ > 0 && Kz > 0 && h > 0 && ψ > 0];
% /. {ρ → 0, θ^2 → 0, ψ^2 → 0, ρ ψ → 0, θ ρ → 0, h → 2 Kz Tan[ΘB], p q → 0, s → 0} // MatrixForm
```

$$\begin{pmatrix} e^{\frac{1}{2} i (2 z (-p \theta + q \psi) + 2 Kz\, z\, \theta \tan[\Theta B])} \tilde{\psi}_{in}[p - Kz\, \theta, q + Kz\, \psi, z] \\ 0 \end{pmatrix}$$

4. Input wave: partially coherent source

direct-space representation of the density matrix, Eq. (9a-b)

```
Clear["Global`*"]; Remove["Global`*"]
ϕ[x_] = e^(-(x-x0)^2/l0^2 + i ϕ0 + i p0 x);
(* j(x1,x2) = ∫_k ⟨x1|ϕ(k)⟩⟨ϕ(k)|x2⟩dk, Cohen-Tannoudji pag. 305, Eq. (28) *)
step1 = ϕ[x1] (ϕ[x2] /. i → -i);
step2 =
  1/Sqrt[2 π σp] Integrate[step1 e^(-p0^2/(2 σp^2)), {p0, -Infinity, +Infinity}, Assumptions → σp > 0];
(* we assume w0 >> l0 *)
step3 = Sqrt[2/π] Sqrt[1/l0^2 + 1/w0^2] Integrate[step2 e^(-2 x0^2/w0^2),
    {x0, -Infinity, +Infinity}, Assumptions → w0 > 0 && l0 > 0] /. l0^2 + w0^2 → w0^2;
Collect[step3[[2]], {x1^2 + x2^2, (x1 - x2)^2}, Together] /. (-1 - l0^2 σp^2)/l0^2 → -1/ℓ0
  (* ℓ0 definition *);
Print["j[x1,x2] = ", Exp[%]] (* Eq. (10b) *);
```

$$j[x1,x2] = e^{-\frac{x1^2+x2^2}{w0^2} - \frac{(x1-x2)^2}{2\,\ell 0}}$$

without assuming w0 >> l0 (redefinitions of w0 and ℓ0)

```
step2 =
  1/(Sqrt[2 π] σp) Integrate[step1 e^(-p0^2/(2 σp^2)), {p0, -Infinity, +Infinity}, Assumptions → σp > 0];
step3 = Sqrt[2/π] Sqrt[1/l0^2 + 1/w0^2]
   Integrate[step2 e^(-2 x0^2/w0^2), {x0, -Infinity, +Infinity}, Assumptions → w0 > 0 && l0 > 0];
(* w0 redefinition and l0 definition *)
Collect[step3[[2]], {x1^2 + x2^2, (x1 - x2)^2}, Together] /.
  {-l0^2 - w0^2 → -w0^2, (-w0^2 - l0^4 σp^2 - l0^2 w0^2 σp^2)/(l0^2 (l0^2 + w0^2)) → 1/ℓ0};
step3 = Exp[%];
Print["j[x1,x2] = ", step3]
(* perfectly coherent state *)
Print["j[x1,x2] = ", Limit[step3, ℓ0 → Infinity]]
```

$j[x1,x2] = e^{-\frac{x1^2+x2^2}{w0^2}+\frac{(x1-x2)^2}{2\,\ell 0}}$

$j[x1,x2] = e^{-\frac{x1^2+x2^2}{w0^2}}$

reciprocal-space representation of the density matrix, Eq. (9c-d)

```
Clear["Global`*"]; Remove["Global`*"]
j[x1_, x2_] = e^(-(x1^2 + x2^2)/w0^2 - (x1 - x2)^2/(2 ℓ0^2));
step1 = Sqrt[2 π] Sqrt[2/w0^2 + 1/ℓ0^2]
   InverseFourierTransform[j[x1, x2], {x2}, {p2}, FourierParameters → {1, -1}];
step2 = Sqrt[1/w0^2 + 1/(w0^2 + 2 ℓ0^2)]/Sqrt[π]
   FullSimplify[FourierTransform[step1, {x1}, {p1}, FourierParameters → {1, -1}]];
% /. w0^2 + ℓ0^2 → w0^2; (* l0 << w0 *)
j̃[p1, p2] == FullSimplify[%] (* Eq. (9d) *)
Print["j̃[p1,p2] =", Limit[step2, ℓ0 → Infinity]] (* Eq. (9e) *);
```

$\tilde{j}[p1, p2] == e^{-\frac{1}{8}(p1-p2)^2 w0^2 - \frac{1}{4}(p1^2+p2^2)\,\ell 0^2}$

$\tilde{j}[p1,p2] = e^{-\frac{1}{4}(p1^2+p2^2)\,w0^2}$

5. Exit waves: coherent source

definitions

```
Clear["Global`*"]; Remove["Global`*"];

U0[p_, q_, z_] = {
    {T[p, z], R[p, z]},
    {R[p, z], T[-p, z]}} ⅇ^(-(ⅈ (p^2 + q^2) z)/(2 Kz)); (* Eq. (4a) *)
F[p_, q_, z_] = {
    {ⅇ^(+ⅈ p z Tan[θB]), 0},
    {0, ⅇ^(-ⅈ p z Tan[θB])}} ⅇ^(-(ⅈ (p^2 + q^2) z)/(2 Kz)); (* Eq. (3c) *)
(* transformation (6b) *)
Mz1[p_, p1_, q_, q1_, z_] = {
    {ⅇ^((ⅈ h (s+θ z))/2) DiracDelta[p1 - p - ρ q + θ Kz, q1 - q - (h ρ)/2 - ψ Kz], 0},
    {0, ⅇ^(-(ⅈ h (s+θ z))/2) DiracDelta[p1 - p - ρ q + θ Kz, q1 - q + (h ρ)/2 - ψ Kz]}
   } ⅇ^(-ⅈ (p z θ-q z ψ)); (* Eq. (6b) *)
(* inverse of the transformation (6b) *)
Mz2[p_, p1_, q_, q1_, z_] = Mz1[p, p1, q, q1, z] /. {s → -s, θ → -θ, ρ → -ρ, ψ → -ψ};

Po = {{1, 0}, {0, 0}};
Ph = {{0, 0}, {0, 1}};

arm1 = F[p, q, zD].Mz2[p, p1, q, q1, Δz + tA].U0[p1, q1, tA].Mz1[p1, p2, q1, q2, Δz].
   F[p2, q2, zA1].Ph.U0[p2, q2, tM1].F[p2, q2, zM1].
   Po.U0[p2, q2, tS].F[p2, q2, zS].{ψ̃in[p2, q2], 0}; (* Eq. (7b) *)

arm2 = F[p, q, zD].Mz2[p, p1, q, q1, Δz + tA].U0[p1, q1, tA].
   F[p1, q1, zA2].Po.U0[p1, q1, tM2].Mz1[p1, p2, q1, q2, Δz - tM2 - zA2].
   F[p2, q2, zM2].Ph.U0[p2, q2, tS].F[p2, q2, zS].{ψ̃in[p2, q2], 0}; (* Eq. (7c) *)

(* the factor of 4 is form δ[p1-p-ρq+θKz,q1-q-hρ/2] =
 2δ[2q-2q1+hρ,-p+p1+Kzθ-qρ] *)
arm1o = arm1〚1〛 / 4
arm1h = arm1〚2〛 / 4
arm2o = arm2〚1〛 / 4
arm2h = arm2〚2〛 / 4
```

$$ⅇ^{-\frac{ⅈ\,(p1^2+q1^2)\,tA}{2\,Kz}-\frac{ⅈ\,(p2^2+q2^2)\,tM1}{2\,Kz}-\frac{ⅈ\,(p2^2+q2^2)\,tS}{2\,Kz}-\frac{ⅈ\,(p2^2+q2^2)\,zA1}{2\,Kz}-\frac{ⅈ\,(p^2+q^2)\,zD}{2\,Kz}-\frac{ⅈ\,(p2^2+q2^2)\,zM1}{2\,Kz}-\frac{ⅈ\,(p2^2+q2^2)\,zS}{2\,Kz}-\frac{1}{2}\,ⅈ\,h\,(s+\Delta z\,\theta)+\frac{1}{2}\,ⅈ\,h\,(-s-(tA+\Delta z)\,\theta)-ⅈ\,(p1\,\Delta z\,\theta-}$$

$$\text{DiracDelta}[-p+p1-Kz\,\theta+q\,\rho,\ -2\,q+2\,q1+h\,\rho+2\,Kz\,\psi]\times$$
$$\text{DiracDelta}[-p1+p2+Kz\,\theta-q1\,\rho,\ -2\,q1+2\,q2+h\,\rho-2\,Kz\,\psi]\times$$
$$R[p1,\ tA]\times R[p2,\ tM1]\times T[p2,\ tS]\ \tilde{\psi}_{in}[p2,\ q2]$$

$$e^{-\frac{i\,(p1^2+q1^2)\,tA}{2\,Kz}-\frac{i\,(p2^2+q2^2)\,tM1}{2\,Kz}-\frac{i\,(p2^2+q2^2)\,tS}{2\,Kz}-\frac{i\,(p2^2+q2^2)\,zA1}{2\,Kz}-\frac{i\,(p^2+q^2)\,zD}{2\,Kz}-\frac{i\,(p2^2+q2^2)\,zM1}{2\,Kz}-\frac{i\,(p2^2+q2^2)\,zS}{2\,Kz}-\frac{1}{2}\,i\,h\,(s+\Delta z\,\theta)-\frac{1}{2}\,i\,h\,(-s-(tA+\Delta z)\,\theta)-i\,(p1\,\Delta z\,\theta-}$$

$$\text{DiracDelta}[-p+p1-Kz\,\theta+q\,\rho,\ 2\,q-2\,q1+h\,\rho-2\,Kz\,\psi]\times$$
$$\text{DiracDelta}[-p1+p2+Kz\,\theta-q1\,\rho,\ -2\,q1+2\,q2+h\,\rho-2\,Kz\,\psi]\times$$
$$R[p2,\ tM1]\times T[-p1,\ tA]\times T[p2,\ tS]\ \tilde{\psi}_{in}[p2,\ q2]$$

$$e^{-\frac{i\,(p1^2+q1^2)\,tA}{2\,Kz}-\frac{i\,(p1^2+q1^2)\,tM2}{2\,Kz}-\frac{i\,(p2^2+q2^2)\,tS}{2\,Kz}-\frac{i\,(p1^2+q1^2)\,zA2}{2\,Kz}-\frac{i\,(p^2+q^2)\,zD}{2\,Kz}-\frac{i\,(p2^2+q2^2)\,zM2}{2\,Kz}-\frac{i\,(p2^2+q2^2)\,zS}{2\,Kz}+\frac{1}{2}\,i\,h\,(-s-(tA+\Delta z)\,\theta)-\frac{1}{2}\,i\,h\,(s+(-tM2-zA2+\Delta z)\,\theta)}$$

$$\text{DiracDelta}[-p+p1-Kz\,\theta+q\,\rho,\ -2\,q+2\,q1+h\,\rho+2\,Kz\,\psi]\times$$
$$\text{DiracDelta}[-p1+p2+Kz\,\theta-q1\,\rho,\ -2\,q1+2\,q2+h\,\rho-2\,Kz\,\psi]\times$$
$$R[p1,\ tM2]\times R[p2,\ tS]\times T[p1,\ tA]\ \tilde{\psi}_{in}[p2,\ q2]$$

$$e^{-\frac{i\,(p1^2+q1^2)\,tA}{2\,Kz}-\frac{i\,(p1^2+q1^2)\,tM2}{2\,Kz}-\frac{i\,(p2^2+q2^2)\,tS}{2\,Kz}-\frac{i\,(p1^2+q1^2)\,zA2}{2\,Kz}-\frac{i\,(p^2+q^2)\,zD}{2\,Kz}-\frac{i\,(p2^2+q2^2)\,zM2}{2\,Kz}-\frac{i\,(p2^2+q2^2)\,zS}{2\,Kz}-\frac{1}{2}\,i\,h\,(-s-(tA+\Delta z)\,\theta)-\frac{1}{2}\,i\,h\,(s+(-tM2-zA2+\Delta z)\,\theta)}$$

$$\text{DiracDelta}[-p+p1-Kz\,\theta+q\,\rho,\ 2\,q-2\,q1+h\,\rho-2\,Kz\,\psi]\times$$
$$\text{DiracDelta}[-p1+p2+Kz\,\theta-q1\,\rho,\ -2\,q1+2\,q2+h\,\rho-2\,Kz\,\psi]\times$$
$$R[p1,\ tA]\times R[p1,\ tM2]\times R[p2,\ tS]\ \tilde{\psi}_{in}[p2,\ q2]$$

final states, Eq. (11a-d)

```
{-2 q + 2 q1 + h ρ + 2 Kz ψ == 0, -p + p1 - Kz θ + q ρ == 0,
   -2 q1 + 2 q2 + h ρ - 2 Kz ψ == 0, -p1 + p2 + Kz θ - q1 ρ == 0};
Expand[Solve[%, {p1, q1, p2, q2}] /. {ρ^2 → 0, ρ ψ → 0}][[1]]
arm1o /. %;(* this is equivalent to the integrations over p2 and p1 *)
ExpandAll[%] /. {ρ^2 → 0, θ^2 → 0, θ ρ → 0, p θ → 0, p ρ → 0, ψ^2 → 0, ψ ρ → 0} /.
   DiracDelta[0, 0]^2 → 1 / 2;
(* omission of the phase terms shared by ψ̃o1 and ψ̃o2 *)
tmp = ExpandAll[
    % e^(-i p (zS+zD) Tan[ΘB]) e^(i h s) e^(-(i h q tA ρ)/(2 Kz)) e^(1/2 i h θ tA) e^(i h Δz θ) e^(i p (zA1-zM1) Tan[ΘB]) e^((-i h q (tM1+tS+zA1+zM1+zS) ρ)/Kz)];
Collect[Part[%, 1, 2], {p, q}, Simplify] /. {tA + tM1 + tS + zA1 + zD + zM1 + zS → zD}
ψ̃o1 == ReplacePart[tmp, 1 → e^Collect[%,zD,Simplify]] (* Eq. (11.a) *)
```

$$\left\{p1\to p+Kz\,\theta-q\,\rho,\ q1\to q-\frac{h\,\rho}{2}-Kz\,\psi,\ p2\to p,\ q2\to q-h\,\rho\right\}$$

$$-\frac{i\,p^2\,zD}{2\,Kz}-\frac{i\,q^2\,zD}{2\,Kz}$$

$$\tilde{\psi}_{o1}==e^{-\frac{i\,(p^2+q^2)\,zD}{2\,Kz}}\ R[p,\ tM1]\times R[p+Kz\,\theta-q\,\rho,\ tA]\times T[p,\ tS]\ \tilde{\psi}_{in}[p,\ q-h\,\rho]$$

```
{-2 q + 2 q1 + h ρ + 2 Kz ψ == 0, -p + p1 - Kz θ + q ρ == 0,
  -2 q1 + 2 q2 + h ρ - 2 Kz ψ == 0, -p1 + p2 + Kz θ - q1 ρ == 0};
Expand[Solve[%, {p1, q1, p2, q2}] /. {ρ^2 → 0, ρ ψ → 0}]⟦1⟧
arm2o /. %;(* this is equivalent to the integrations over p2 and p1 *)
ExpandAll[%] /. {ρ^2 → 0, θ^2 → 0, θ ρ → 0, p θ → 0, p ρ → 0, ψ^2 → 0, ψ ρ → 0} /.
  DiracDelta[0, 0]^2 → 1 / 2;
(* omission of the shared phase terms, ψ̃o1 and ψ̃o2 *)
tmp = ExpandAll[
   % ⅇ^(-ⅈ p (zS+zD) Tan[ΘB]) ⅇ^(ⅈ h s) ⅇ^(1/2 ⅈ h θ tA) ⅇ^(-ⅈ h q tA ρ/(2 Kz)) ⅇ^(ⅈ h Δz θ) ⅇ^(ⅈ p (zA1-zM1) Tan[ΘB]) ⅇ^(-ⅈ h q (tM1+tS+zA1+zM1+zS) ρ/Kz)];
Collect[Part[%, 1, 2], {p, q}, Simplify] /. {tA + tM2 + tS + zA2 + zD + zM2 + zS → zD,
   (zA1 + zA2 - zM1 - zM2) Tan[ΘB] → Δx, Tan[ΘB] → h / (2 Kz)};
Collect[%, {p, q, ρ}, Simplify] /.
 {(tM2 + 2 zA2) θ → 2 s0, -ⅈ h (2 tM1 - tM2 + 2 (zA1 + zM1 - zM2)) ρ/(2 Kz) → -ⅈ Δy}
ψ̃o2 == ReplacePart[tmp, 1 → ⅇ^Collect[%,zD,Simplify]](* Eq. (11.b) *)
```

$$\left\{p1 \to p + Kz\,\theta - q\,\rho,\ q1 \to q - \frac{h\,\rho}{2} - Kz\,\psi,\ p2 \to p - Kz\,\rho\,\psi,\ q2 \to q - h\,\rho\right\}$$

$$i\,h\,s0 - \frac{i\,p^2\,zD}{2\,Kz} - \frac{i\,q^2\,zD}{2\,Kz} + i\,p\,\Delta x - i\,q\,\Delta y$$

$$\tilde{\psi}_{o2} == e^{-\frac{i\,(p^2+q^2)\,zD}{2\,Kz} + i\,(h\,s0 + p\,\Delta x - q\,\Delta y)}\ R[p, tS] \times R[p + Kz\,\theta - q\,\rho, tM2] \times T[p + Kz\,\theta - q\,\rho, tA]\ \tilde{\psi}_{in}[p, q - h\,\rho]$$

```
{2 q - 2 q1 + h ρ - 2 Kz ψ == 0, -p + p1 - Kz θ + q ρ == 0,
  -2 q1 + 2 q2 + h ρ - 2 Kz ψ == 0, -p1 + p2 + Kz θ - q1 ρ == 0};
Expand[Solve[%, {p1, q1, p2, q2}] /. {ρ^2 → 0, ρ ψ → 0}]⟦1⟧
arm1h /. %;(* integrations over p2 and p1 *)
ExpandAll[%] /. {ρ^2 → 0, θ^2 → 0, θ ρ → 0, p θ → 0, p ρ → 0, ψ^2 → 0, ψ ρ → 0} /.
  DiracDelta[0, 0]^2 → 1 / 2;
(* omission of the shared phase terms, ψ̃h1 and ψ̃h2 *)
tmp = ExpandAll[% ⅇ^(-ⅈ p (zS-zD) Tan[ΘB]) ⅇ^(-1/2 ⅈ h θ tA) ⅇ^(ⅈ h q tA ρ/(2 Kz)) ⅇ^(ⅈ p (zA1-zM1) Tan[ΘB])];
Collect[Part[%, 1, 2], {p, q}, Simplify] /. {tA + tM1 + tS + zA1 + zD + zM1 + zS → zD}
ψ̃h1 == ReplacePart[tmp, 1 → ⅇ^Collect[%,zD,Simplify]](* Eq. (11.c) *)
```

$$\left\{p1 \to p + Kz\,\theta - q\,\rho,\ q1 \to q + \frac{h\,\rho}{2} - Kz\,\psi,\ p2 \to p - Kz\,\rho\,\psi,\ q2 \to q\right\}$$

$$-\frac{i\,p^2\,zD}{2\,Kz} - \frac{i\,q^2\,zD}{2\,Kz}$$

$$\tilde{\psi}_{h1} == e^{-\frac{i\,(p^2+q^2)\,zD}{2\,Kz}}\ R[p, tM1] \times T[p, tS] \times T[-p - Kz\,\theta + q\,\rho, tA]\ \tilde{\psi}_{in}[p, q]$$

```
{2 q - 2 q1 + h ρ - 2 Kz ψ == 0, -p + p1 - Kz θ + q ρ == 0,
  -2 q1 + 2 q2 + h ρ - 2 Kz ψ == 0, -p1 + p2 + Kz θ - q1 ρ == 0};
Expand[Solve[%, {p1, q1, p2, q2}] /. {ρ^2 → 0, ρ ψ → 0}] [[1]]
arm2h /. %;(* integrations over p2 and p1 *)
ExpandAll[%] /. {ρ^2 → 0, θ^2 → 0, θ ρ → 0, p θ → 0, p ρ → 0, ψ^2 → 0, ψ ρ → 0} /.
  DiracDelta[0, 0]^2 → 1 / 2;
(* omission of the shared phase terms, ψ̃h1 and ψ̃h2 *)
```

$$\text{tmp} = \text{ExpandAll}\left[\% \, e^{-i\, p\, (zS-zD)\, \text{Tan}[\Theta B]}\, e^{-\frac{1}{2}\, i\, h\, \theta\, tA}\, e^{\frac{i\, h\, q\, tA\, \rho}{2\, Kz}}\, e^{i\, p\, (zA1-zM1)\, \text{Tan}[\Theta B]}\right];$$

```
Simplify[#] & /@ Collect[Part[%, 1, 2], {p, q, θ, ρ}] /.
  {tA + tM2 + tS + zA2 + zD + zM2 + zS → zD,
   (zA1 + zA2 - zM1 - zM2) Tan[ΘB] → Δx, Tan[ΘB] → h / (2 Kz)};
```

$$\text{Collect}[\%, \{p, q\}, \text{Simplify}] \;/.\; \left\{(tM2 + 2\, zA2)\, \theta \to 2\, s0,\; -\frac{i\, h\, q\, (tM2 + 2\, zA2)\, \rho}{2\, Kz} \to -i\, q\, \Delta y\right\}$$

$$\tilde{\psi}_{h2} == \text{ReplacePart}\left[\text{tmp}, 1 \to e^{\text{Collect}[\%, zD, \text{Simplify}]}\right] \text{(* Eq. (11.d) *)}$$

$$\left\{p1 \to p + Kz\, \theta - q\, \rho,\; q1 \to q + \frac{h\, \rho}{2} - Kz\, \psi,\; p2 \to p - Kz\, \rho\, \psi,\; q2 \to q\right\}$$

$$i\, h\, s0 - \frac{i\, p^2\, zD}{2\, Kz} - \frac{i\, q^2\, zD}{2\, Kz} + i\, p\, \Delta x - i\, q\, \Delta y$$

$$\tilde{\psi}_{h2} == e^{-\frac{i\, (p^2+q^2)\, zD}{2\, Kz} + i\, (h\, s0 + p\, \Delta x - q\, \Delta y)}\, R[p, tS] \times R[p + Kz\, \theta - q\, \rho, tA] \times R[p + Kz\, \theta - q\, \rho, tM2]\, \tilde{\psi}_{in}[p, q]$$

6. Exit waves: partially coherent source

definitions

```
Clear["Global`*"]; Remove["Global`*"];

(* crystal diffraction, Laue *)
U0[p_, q_, z_] = {
    {T[p, z], R[p, z]},
    {R[p, z], T[-p, z]}} ⅇ^(-ⅈ (p^2 + q^2) z/(2 Kz)); (* Eq. (4a) *)
(* free-space propagation *)
F[p_, q_, z_] = {
    {ⅇ^(+ⅈ p z Tan[ΘB]), 0},
    {0, ⅇ^(-ⅈ p z Tan[ΘB])}} ⅇ^(-ⅈ (p^2 + q^2) z/(2 Kz)); (* Eq. (3c) *)
(* transformation (6b) *)
Mz1[p_, p1_, q_, q1_, z_] = {
    {ⅇ^(ⅈ h (s+θ z)/2) DiracDelta[p1 - p - ρ q + θ Kz, q1 - q - h ρ/2 - ψ Kz], 0},
    {0, ⅇ^(-ⅈ h (s+θ z)/2) DiracDelta[p1 - p - ρ q + θ Kz, q1 - q + h ρ/2 - ψ Kz]}
  } ⅇ^(-ⅈ (p z θ-q z ψ)); (* Eq. (6b) *)
(* inverse of the transformation (6b) *)
Mz2[p_, p1_, q_, q1_, z_] = Mz1[p, p1, q, q1, z] /. {s → -s, θ → -θ, ρ → -ρ, ψ → -ψ};
(* o and h projectors *)
Po = {{1, 0}, {0, 0}};
Ph = {{0, 0}, {0, 1}};

(* ⟨p,q|X1 Po|p1,q1⟩, to be integrated over p2 and q2, see Eq. (19) *)
arm1 = F[p, q, zDA].Mz2[p, p2, q, q2, Δz + tA].U0[p2, q2, tA].Mz1[p2, p1, q2, q1, Δz].
   F[p1, q1, zA1].Ph.U0[p1, q1, tM1].F[p1, q1, zM1].Po.U0[p1, q1, tS].F[p1, q1, zS].Po;
(* ⟨p,q|X2 Po|p1,q1⟩, to be integrated over p2 and q2, see Eq. (19) *)
arm2 = F[p, q, zDA].Mz2[p, p2, q, q2, Δz + tA].U0[p2, q2, tA].
   F[p2, q2, zA2].Po.U0[p2, q2, tM2].Mz1[p2, p1, q2, q1, Δz - tM2 - zA2].
   F[p1, q1, zM2].Ph.U0[p1, q1, tS].F[p1, q1, zS].Po;

(* the factor of 4 is form δ[p1-p-ρ q+θ Kz,q1-q-hρ/2] =
 2δ[2 q-2 q1+h ρ,-p+p1+Kz θ-q ρ] *)
X1Poo = arm1〚1, 1〛 / 4
X1Poh = arm1〚2, 1〛 / 4
X2Poo = arm2〚1, 1〛 / 4
X2Poh = arm2〚2, 1〛 / 4
```

$$e^{-\frac{i(p2^2+q2^2)\,tA}{2\,Kz}-\frac{i(p1^2+q1^2)\,tM1}{2\,Kz}-\frac{i(p1^2+q1^2)\,tS}{2\,Kz}-\frac{i(p1^2+q1^2)\,zA1}{2\,Kz}-\frac{i(p^2+q^2)\,zDA}{2\,Kz}-\frac{i(p1^2+q1^2)\,zM1}{2\,Kz}-\frac{i(p1^2+q1^2)\,zS}{2\,Kz}-\frac{1}{2}\,i\,h\,(s+\Delta z\,\theta)+\frac{1}{2}\,i\,h\,(-s-(tA+\Delta z)\,\theta)-i\,(p2\,\Delta z\,\theta\cdot}$$

```
 DiracDelta[-p + p2 - Kz θ + q ρ, -2 q + 2 q2 + h ρ + 2 Kz ψ] ×
 DiracDelta[p1 - p2 + Kz θ - q2 ρ, 2 q1 - 2 q2 + h ρ - 2 Kz ψ] × R[p1, tM1] × R[p2, tA] × T[p1, tS]
```

$$e^{-\frac{i\,(p2^2+q2^2)\,tA}{2\,Kz}-\frac{i\,(p1^2+q1^2)\,tM1}{2\,Kz}-\frac{i\,(p1^2+q1^2)\,tS}{2\,Kz}-\frac{i\,(p1^2+q1^2)\,zA1}{2\,Kz}-\frac{i\,(p^2+q^2)\,zDA}{2\,Kz}-\frac{i\,(p1^2+q1^2)\,zM1}{2\,Kz}-\frac{i\,(p1^2+q1^2)\,zS}{2\,Kz}-\frac{1}{2}\,i\,h\,(s+\Delta z\,\theta)-\frac{1}{2}\,i\,h\,(-s-(tA+\Delta z)\,\theta)-i\,(p2\,\Delta z\,\theta\cdot}$$

$$\text{DiracDelta}[-p+p2-Kz\,\theta+q\,\rho,\ 2\,q-2\,q2+h\,\rho-2\,Kz\,\psi]\times$$
$$\text{DiracDelta}[p1-p2+Kz\,\theta-q2\,\rho,\ 2\,q1-2\,q2+h\,\rho-2\,Kz\,\psi]\times R[p1,\ tM1]\times T[p1,\ tS]\times T[-p2,\ tA]$$

$$e^{-\frac{i\,(p2^2+q2^2)\,tA}{2\,Kz}-\frac{i\,(p2^2+q2^2)\,tM2}{2\,Kz}-\frac{i\,(p1^2+q1^2)\,tS}{2\,Kz}-\frac{i\,(p2^2+q2^2)\,zA2}{2\,Kz}-\frac{i\,(p^2+q^2)\,zDA}{2\,Kz}-\frac{i\,(p1^2+q1^2)\,zM2}{2\,Kz}-\frac{i\,(p1^2+q1^2)\,zS}{2\,Kz}+\frac{1}{2}\,i\,h\,(-s-(tA+\Delta z)\,\theta)-\frac{1}{2}\,i\,h\,(s+(-tM2-zA2+\Delta z)\,\theta}$$

$$\text{DiracDelta}[-p+p2-Kz\,\theta+q\,\rho,\ -2\,q+2\,q2+h\,\rho+2\,Kz\,\psi]\times$$
$$\text{DiracDelta}[p1-p2+Kz\,\theta-q2\,\rho,\ 2\,q1-2\,q2+h\,\rho-2\,Kz\,\psi]\times R[p1,\ tS]\times R[p2,\ tM2]\times T[p2,\ tA]$$

$$e^{-\frac{i\,(p2^2+q2^2)\,tA}{2\,Kz}-\frac{i\,(p2^2+q2^2)\,tM2}{2\,Kz}-\frac{i\,(p1^2+q1^2)\,tS}{2\,Kz}-\frac{i\,(p2^2+q2^2)\,zA2}{2\,Kz}-\frac{i\,(p^2+q^2)\,zDA}{2\,Kz}-\frac{i\,(p1^2+q1^2)\,zM2}{2\,Kz}-\frac{i\,(p1^2+q1^2)\,zS}{2\,Kz}-\frac{1}{2}\,i\,h\,(-s-(tA+\Delta z)\,\theta)-\frac{1}{2}\,i\,h\,(s+(-tM2-zA2+\Delta z)\,\theta}$$

$$\text{DiracDelta}[-p+p2-Kz\,\theta+q\,\rho,\ 2\,q-2\,q2+h\,\rho-2\,Kz\,\psi]\times$$
$$\text{DiracDelta}[p1-p2+Kz\,\theta-q2\,\rho,\ 2\,q1-2\,q2+h\,\rho-2\,Kz\,\psi]\times R[p1,\ tS]\times R[p2,\ tA]\times R[p2,\ tM2]$$

Appendix C

calculation of $(XPo)_i^{nn}$[p, q, p1, q1]in Eq. (19)

the integration over p2 is because of the transformation (6b)

the phase terms shared by $(XPo)_1^{nn}$ and $(XPo)_2^{nn}$ are omitted

```
{-2 q + 2 q2 + h ρ + 2 Kz ψ == 0, -p + p2 - Kz θ + q ρ == 0,
  2 q1 - 2 q2 + h ρ - 2 Kz ψ == 0, p1 - p2 + Kz θ - q2 ρ == 0};
rule = Expand[Solve[%, {p2, q2, p1, q1}] /. {ρ^2 → 0, ρ ψ → 0}][[1]]
X1Poo /. %(* integrations over p2 *);
ExpandAll[% / 2] /. {ρ^2 → 0, θ^2 → 0, ψ^2 → 0, θ ρ → 0, ρ ψ → 0, p ρ → 0, p θ → 0} /.
  DiracDelta[0, 0]^2 → DiracDelta[p1 - (p1 /. rule), q1 - (q1 /. rule)];
(* omission of the shared phase terms, Xo͂o1 and Xo͂o2 *)
tmp = ExpandAll[
   % e^(-i p (zS+zDA) Tan[ΘB]) e^(i h s) e^(-(i h q tA ρ)/(2 Kz)) e^(1/2 i h θ tA) e^(i h Δz θ) e^(i p (zA1-zM1) Tan[ΘB]) e^((-i h q (tM1+tS+zA1+zM1+zS) ρ)/Kz)];
Collect[Part[%, 1, 2], {p, q}, Simplify] /. {tA + tM1 + tS + zA1 + zDA + zM1 + zS → zD}
Xo͂o1 = ReplacePart[tmp, 1 → e^Collect[%,zD,Simplify]];
Print["Xo͂o1 = ", Xo͂o1]
```

$$\left\{p2\to p+Kz\,\theta-q\,\rho,\ q2\to q-\frac{h\,\rho}{2}-Kz\,\psi,\ p1\to p,\ q1\to q-h\,\rho\right\}$$

$$-\frac{i\,p^2\,zD}{2\,Kz}-\frac{i\,q^2\,zD}{2\,Kz}$$

$$\text{X}\tilde{\text{o}}\text{o1} = e^{-\frac{i\,(p^2+q^2)\,zD}{2\,Kz}}\ \text{DiracDelta}[-p+p1,\ -q+q1+h\,\rho]\times R[p,\ tM1]\times R[p+Kz\,\theta-q\,\rho,\ tA]\times T[p,\ tS]$$

```
{-p + p2 - Kz θ + q ρ == 0, -2 q + 2 q2 + h ρ + 2 Kz ψ == 0,
  p1 - p2 + Kz θ - q2 ρ == 0, 2 q1 - 2 q2 + h ρ - 2 Kz ψ == 0};
rule = Expand[Solve[%, {p2, q2, p1, q1}] /. {ρ^2 → 0, ρ ψ → 0}][[1]]
X2Poo /. %;(* integrations over p2 *);
ExpandAll[% / 2] /. {ρ^2 → 0, θ^2 → 0, ψ^2 → 0, θ ρ → 0, ρ ψ → 0, p ρ → 0, p θ → 0} /.
  DiracDelta[0, 0]^2 → DiracDelta[p1 - (p1 /. rule), q1 - (q1 /. rule)];
(* omission of the shared phase terms, Xõo1 and Xõo2 *)
tmp = ExpandAll[
   % e^(-i p (zS+zDA) Tan[ΘB]) e^(i h s) e^(-(i h q tA ρ)/(2 Kz)) e^(1/2 i h θ tA) e^(i h Δz θ) e^(i p (zA1-zM1) Tan[ΘB]) e^((-i h q (tM1+tS+zA1+zM1+zS) ρ)/Kz)];
Collect[Part[%, 1, 2], {p, q}, Simplify] /. {tA + tM2 + tS + zA2 + zDA + zM2 + zS → zD,
   (zA1 + zA2 - zM1 - zM2) Tan[ΘB] → Δx, Tan[ΘB] → h / (2 Kz)};
Collect[%, {p, q, ρ}, Simplify] /.
 {(tM2 + 2 zA2) θ → 2 s0, -(i h (2 tM1 - tM2 + 2 (zA1 + zM1 - zM2)) ρ)/(2 Kz) → -i Δy}
Xõo2 = ReplacePart[tmp, 1 → e^Collect[%,zD,Simplify]];
Print["Xõo2 = ", Xõo2]
```

$$\left\{p2 \to p + Kz\,\theta - q\,\rho,\ q2 \to q - \frac{h\,\rho}{2} - Kz\,\psi,\ p1 \to p,\ q1 \to q - h\,\rho\right\}$$

$$i\,h\,s0 - \frac{i\,p^2\,zD}{2\,Kz} - \frac{i\,q^2\,zD}{2\,Kz} + i\,p\,\Delta x - i\,q\,\Delta y$$

$$X\tilde{o}o2 = e^{-\frac{i\,(p^2+q^2)\,zD}{2\,Kz} + i\,(h\,s0 + p\,\Delta x - q\,\Delta y)}\ \mathrm{DiracDelta}[-p + p1,\ -q + q1 + h\,\rho] \times R[p,\ tS] \times R[p + Kz\,\theta - q\,\rho,\ tM2] \times T[p + Kz\,\theta - q\,\rho,\ tA]$$

```
{-p + p2 - Kz θ + q ρ == 0, 2 q - 2 q2 + h ρ - 2 Kz ψ == 0,
  p1 - p2 + Kz θ - q2 ρ == 0, 2 q1 - 2 q2 + h ρ - 2 Kz ψ == 0};
rule = Expand[Solve[%, {p2, q2, p1, q1}] /. {ρ^2 → 0, ρ ψ → 0}][[1]]
X1Poh /. %;(* integrations over p2 *);
ExpandAll[% / 2] /. {ρ^2 → 0, θ^2 → 0, ψ^2 → 0, θ ρ → 0, ρ ψ → 0, p ρ → 0, p θ → 0} /.
  DiracDelta[0, 0]^2 → DiracDelta[p1 - (p1 /. rule), q1 - (q1 /. rule)];
(* omission of the shared phase terms, Xh̃h1 and Xh̃h2 *)
tmp = ExpandAll[% e^(-i p (zS-zDA) Tan[ΘB]) e^(-1/2 i h θ tA) e^((i h q tA ρ)/(2 Kz)) e^(i p (zA1-zM1) Tan[ΘB])];
Collect[Part[%, 1, 2], {p, q}, Simplify] /. {tA + tM1 + tS + zA1 + zDA + zM1 + zS → zD}
Xh̃h1 = ReplacePart[tmp, 1 → e^Collect[%,zD,Simplify]];
Print["Xh̃h1 = ", Xh̃h1]
```

$$\left\{p2 \to p + Kz\,\theta - q\,\rho,\ q2 \to q + \frac{h\,\rho}{2} - Kz\,\psi,\ p1 \to p,\ q1 \to q\right\}$$

$$-\frac{i\,p^2\,zD}{2\,Kz} - \frac{i\,q^2\,zD}{2\,Kz}$$

$$X\tilde{h}h1 = e^{-\frac{i\,(p^2+q^2)\,zD}{2\,Kz}}\ \mathrm{DiracDelta}[-p + p1,\ -q + q1] \times R[p,\ tM1] \times T[p,\ tS] \times T[-p - Kz\,\theta + q\,\rho,\ tA]$$

```
{-p + p2 - Kz θ + q ρ == 0, 2 q - 2 q2 + h ρ - 2 Kz ψ == 0,
   p1 - p2 + Kz θ - q2 ρ == 0, 2 q1 - 2 q2 + h ρ - 2 Kz ψ == 0};
rule = Expand[Solve[%, {p2, q2, p1, q1}] /. {ρ^2 → 0, ρ ψ → 0}][[1]]
X2Poh /. %(* integrations over p2 *);
ExpandAll[% / 2] /. {ρ^2 → 0, θ^2 → 0, ψ^2 → 0, θ ρ → 0, ρ ψ → 0, p ρ → 0, p θ → 0} /.
   DiracDelta[0, 0]^2 → DiracDelta[p1 - (p1 /. rule), q1 - (q1 /. rule)];
(* omission of the shared phase terms, Xh̃h1 and Xh̃h2 *)
tmp = ExpandAll[% ⅇ^(-ⅈ p (zS-zDA) Tan[ΘB]) ⅇ^(-1/2 ⅈ h θ tA) ⅇ^(ⅈ h q tA ρ/(2 Kz)) ⅇ^(ⅈ p (zA1-zM1) Tan[ΘB])];
Simplify[#] & /@ Collect[Part[%, 1, 2], {p, q, θ, ρ}] /.
   {tA + tM2 + tS + zA2 + zDA + zM2 + zS → zD,
    (zA1 + zA2 - zM1 - zM2) Tan[ΘB] → Δx, Tan[ΘB] → h / (2 Kz)};
Collect[%, {p, q}, Simplify] /. {(tM2 + 2 zA2) θ → 2 s0, - (ⅈ h q (tM2 + 2 zA2) ρ)/(2 Kz) → -ⅈ q Δy}
Xh̃h2 = ReplacePart[tmp, 1 → ⅇ^Collect[%,zD,Simplify]];
Print["Xh̃h2 = ", Xh̃h2]
```

$$\left\{p2 \to p + Kz\,\theta - q\,\rho,\ q2 \to q + \frac{h\,\rho}{2} - Kz\,\psi,\ p1 \to p,\ q1 \to q\right\}$$

$$i\,h\,s0 - \frac{i\,p^2\,zD}{2\,Kz} - \frac{i\,q^2\,zD}{2\,Kz} + i\,p\,\Delta x - i\,q\,\Delta y$$

$$\text{Xh̃h2} = e^{-\frac{i\,(p^2+q^2)\,zD}{2\,Kz} + i\,(h\,s0 + p\,\Delta x - q\,\Delta y)}\,\text{DiracDelta}[-p + p1, -q + q1] \times R[p, tS] \times R[p + Kz\,\theta - q\,\rho, tA] \times R[p + Kz\,\theta - q\,\rho, tM2]$$

Appendix C
calculation of $(XPo)_{i}^{*nn}[p, q, p2, q2]$ in Eq. (19)

```
Xõo1* = Xõo1 /. Exp[x_] → Exp[-x] /. {p1 → p2, q1 → q2} /.
   {R[x_, y_] → R*[x, y], T[x_, y_] → T*[x, y]}
Xõo2* = Xõo2 /. Exp[x_] → Exp[-x] /. {p1 → p2, q1 → q2} /.
   {R[x_, y_] → R*[x, y], T[x_, y_] → T*[x, y]}
Xh̃h1* = Xh̃h1 /. Exp[x_] → Exp[-x] /. {p1 → p2, q1 → q2} /.
   {R[x_, y_] → R*[x, y], T[x_, y_] → T*[x, y]}
Xh̃h2* = Xh̃h2 /. Exp[x_] → Exp[-x] /. {p1 → p2, q1 → q2} /.
   {R[x_, y_] → R*[x, y], T[x_, y_] → T*[x, y]}
```

$$e^{\frac{i\,(p^2+q^2)\,zD}{2\,Kz}}\,\text{DiracDelta}[-p + p2, -q + q2 + h\,\rho]\,R^*[p, tM1]\,R^*[p + Kz\,\theta - q\,\rho, tA]\,T^*[p, tS]$$

$$e^{\frac{i\,(p^2+q^2)\,zD}{2\,Kz} - i\,(h\,s0 + p\,\Delta x - q\,\Delta y)}\,\text{DiracDelta}[-p + p2, -q + q2 + h\,\rho]\,R^*[p, tS]\,R^*[p + Kz\,\theta - q\,\rho, tM2]\,T^*[p + Kz\,\theta - q\,\rho, tA]$$

$$e^{\frac{i\,(p^2+q^2)\,zD}{2\,Kz}}\,\text{DiracDelta}[-p + p2, -q + q2]\,R^*[p, tM1]\,T^*[p, tS]\,T^*[-p - Kz\,\theta + q\,\rho, tA]$$

$$e^{\frac{i\,(p^2+q^2)\,zD}{2\,Kz} - i\,(h\,s0 + p\,\Delta x - q\,\Delta y)}\,\text{DiracDelta}[-p + p2, -q + q2]\,R^*[p, tS]\,R^*[p + Kz\,\theta - q\,\rho, tA]\,R^*[p + Kz\,\theta - q\,\rho, tM2]$$

Appendix C

calculation of $\tilde{j}_o^{ij}$[pa, qa, pb, qb)]

```
(X̃oo1 /. {p → pa, q → qa}) (X̃oo1* /. {p → pb, q → qb}) jin[p1, q1, p2, q2];
tmp = Integrate[%, {p1, -Infinity, +Infinity}, {q1, -Infinity, +Infinity},
   {p2, -Infinity, +Infinity}, {q2, -Infinity, +Infinity}, Assumptions → pa ∈ ℝ];
Collect[tmp[[1, 2]], zD, Simplify]
j̃o11 == ReplacePart[tmp, {1, 2} → %] (* Eq. (C.1b) *)
```

$$-\frac{\mathrm{i}\,(pa^2 - pb^2 + qa^2 - qb^2)\,zD}{2\,Kz}$$

$$\tilde{jo}11 == e^{-\frac{\mathrm{i}\,(pa^2-pb^2+qa^2-qb^2)\,zD}{2\,Kz}}\,jin[pa,\,qa - h\,\rho,\,pb,\,qb - h\,\rho] \times R[pa,\,tM1] \times R[pa + Kz\,\Theta - qa\,\rho,\,tA] \times T[pa,\,tS]\,R^*[pb,\,tM1]\,R^*[pb + Kz\,\Theta - qb\,\rho,\,tA]\,T^*[pb,\,tS]$$

```
(X̃oo2 /. {p → pa, q → qa}) (X̃oo2* /. {p → pb, q → qb}) jin[p1, q1, p2, q2];
tmp = Integrate[%, {p1, -Infinity, +Infinity}, {q1, -Infinity, +Infinity},
   {p2, -Infinity, +Infinity}, {q2, -Infinity, +Infinity}, Assumptions → pa ∈ ℝ];
Collect[tmp[[1, 2]], {Δx, zD}, Simplify]
j̃o22 == ReplacePart[tmp, {1, 2} → %] (* Eq. (C.1c) *)
```

$$-\frac{\mathrm{i}\,(pa^2 - pb^2 + qa^2 - qb^2)\,zD}{2\,Kz} + \mathrm{i}\,(pa - pb)\,\Delta x - \mathrm{i}\,(qa - qb)\,\Delta y$$

$$\tilde{jo}22 == e^{-\frac{\mathrm{i}\,(pa^2-pb^2+qa^2-qb^2)\,zD}{2\,Kz} + \mathrm{i}\,(pa-pb)\,\Delta x - \mathrm{i}\,(qa-qb)\,\Delta y}\,jin[pa,\,qa - h\,\rho,\,pb,\,qb - h\,\rho] \times R[pa,\,tS] \times R[pa + Kz\,\Theta - qa\,\rho,\,tM2] \times T[pa + Kz\,\Theta - qa\,\rho,\,tA]\,R^*[pb,\,tS]\,R^*[pb + Kz\,\Theta - qb\,\rho,\,tM2]\,T^*[pb + Kz\,\Theta - qb\,\rho,\,tA]$$

```
(X̃oo1 /. {p → pa, q → qa}) (X̃oo2* /. {p → pb, q → qb}) jin[p1, q1, p2, q2];
tmp = Integrate[%, {p1, -Infinity, +Infinity}, {q1, -Infinity, +Infinity},
   {p2, -Infinity, +Infinity}, {q2, -Infinity, +Infinity}, Assumptions → pa ∈ ℝ];
Collect[tmp[[1, 2]], zD, Simplify]
j̃o12 == ReplacePart[tmp, {1, 2} → %] (* Eq. (C.1d) *)
```

$$-\frac{\mathrm{i}\,(pa^2 - pb^2 + qa^2 - qb^2)\,zD}{2\,Kz} - \mathrm{i}\,(h\,s0 + pb\,\Delta x - qb\,\Delta y)$$

$$\tilde{jo}12 == e^{-\frac{\mathrm{i}\,(pa^2-pb^2+qa^2-qb^2)\,zD}{2\,Kz} - \mathrm{i}\,(h\,s0+pb\,\Delta x-qb\,\Delta y)}\,jin[pa,\,qa - h\,\rho,\,pb,\,qb - h\,\rho] \times R[pa,\,tM1] \times R[pa + Kz\,\Theta - qa\,\rho,\,tA] \times T[pa,\,tS]\,R^*[pb,\,tS]\,R^*[pb + Kz\,\Theta - qb\,\rho,\,tM2]\,T^*[pb + Kz\,\Theta - qb\,\rho,\,tA]$$

```
(X̃oo2 /. {p → pa, q → qa}) (X̃oo1* /. {p → pb, q → qb}) jin[p1, q1, p2, q2];
tmp = Integrate[%, {p1, -Infinity, +Infinity}, {q1, -Infinity, +Infinity},
   {p2, -Infinity, +Infinity}, {q2, -Infinity, +Infinity}, Assumptions → pa ∈ ℝ];
Collect[tmp[[1, 2]], zD, Simplify]
j̃o21 == ReplacePart[tmp, {1, 2} → %] (* Eq. (C.1e) *)
```

$$\frac{\mathrm{i}\,(-pa^2 + pb^2 - qa^2 + qb^2)\,zD}{2\,Kz} + \mathrm{i}\,(h\,s0 + pa\,\Delta x - qa\,\Delta y)$$

$$\tilde{jo}21 == e^{\frac{\mathrm{i}\,(-pa^2+pb^2-qa^2+qb^2)\,zD}{2\,Kz} + \mathrm{i}\,(h\,s0+pa\,\Delta x-qa\,\Delta y)}\,jin[pa,\,qa - h\,\rho,\,pb,\,qb - h\,\rho] \times R[pa,\,tS] \times R[pa + Kz\,\Theta - qa\,\rho,\,tM2] \times T[pa + Kz\,\Theta - qa\,\rho,\,tA]\,R^*[pb,\,tM1]\,R^*[pb + Kz\,\Theta - qb\,\rho,\,tA]\,T^*[pb,\,tS]$$

Appendix C
calculation of j_h^{ij}[pa, qa, pb, qb)]

```
(Xh̃h1 /. {p → pa, q → qa}) (Xh̃h1* /. {p → pb, q → qb}) jin[p1, q1, p2, q2];
tmp = Integrate[%, {p1, -Infinity, +Infinity}, {q1, -Infinity, +Infinity},
    {p2, -Infinity, +Infinity}, {q2, -Infinity, +Infinity}, Assumptions → pa ∈ ℝ];
Collect[tmp⟦1, 2⟧, zD, Simplify]
jh̃11 == ReplacePart[tmp, {1, 2} → %] (* Eq. (C.1f) *)
```

$$-\frac{i\left(pa^2-pb^2+qa^2-qb^2\right)zD}{2\,Kz}$$

$$j\tilde{h}11 == e^{-\frac{i\left(pa^2-pb^2+qa^2-qb^2\right)zD}{2\,Kz}}\,jin[pa, qa, pb, qb]\times R[pa, tM1]\times T[pa, tS]\times T[-pa-Kz\,\theta+qa\,\rho, tA]\,R^*[pb, tM1]\,T^*[pb, tS]\,T^*[-pb-Kz\,\theta+qb\,\rho, tA]$$

```
(Xh̃h2 /. {p → pa, q → qa}) (Xh̃h2* /. {p → pb, q → qb}) jin[p1, q1, p2, q2];
tmp = Integrate[%, {p1, -Infinity, +Infinity}, {q1, -Infinity, +Infinity},
    {p2, -Infinity, +Infinity}, {q2, -Infinity, +Infinity}, Assumptions → pa ∈ ℝ];
Collect[tmp⟦1, 2⟧, {Δx, zD}, Simplify]
jh̃22 == ReplacePart[tmp, {1, 2} → %] (* Eq. (C.1g) *)
```

$$-\frac{i\left(pa^2-pb^2+qa^2-qb^2\right)zD}{2\,Kz}+i\,(pa-pb)\,\Delta x+i\,(-qa+qb)\,\Delta y$$

$$j\tilde{h}22 == e^{-\frac{i\left(pa^2-pb^2+qa^2-qb^2\right)zD}{2\,Kz}+i\,(pa-pb)\,\Delta x+i\,(-qa+qb)\,\Delta y}\,jin[pa, qa, pb, qb]\times R[pa, tS]\times R[pa+Kz\,\theta-qa\,\rho, tA]\times R[pa+Kz\,\theta-qa\,\rho, tM2]\,R^*[pb, tS]\,R^*[pb+Kz\,\theta-qb\,\rho, tA]\,R^*[pb+Kz\,\theta-qb\,\rho, tM2]$$

```
(Xh̃h1 /. {p → pa, q → qa}) (Xh̃h2* /. {p → pb, q → qb}) jin[p1, q1, p2, q2];
tmp = Integrate[%, {p1, -Infinity, +Infinity}, {q1, -Infinity, +Infinity},
    {p2, -Infinity, +Infinity}, {q2, -Infinity, +Infinity}, Assumptions → pa ∈ ℝ];
Collect[tmp⟦1, 2⟧, zD, Simplify]
jh̃12 == ReplacePart[tmp, {1, 2} → %] (* Eq. (C.1h) *)
```

$$-\frac{i\left(pa^2-pb^2+qa^2-qb^2\right)zD}{2\,Kz}-i\,(h\,s0+pb\,\Delta x-qb\,\Delta y)$$

$$j\tilde{h}12 == e^{-\frac{i\left(pa^2-pb^2+qa^2-qb^2\right)zD}{2\,Kz}-i\,(h\,s0+pb\,\Delta x-qb\,\Delta y)}\,jin[pa, qa, pb, qb]\times R[pa, tM1]\times T[pa, tS]\times T[-pa-Kz\,\theta+qa\,\rho, tA]\,R^*[pb, tS]\,R^*[pb+Kz\,\theta-qb\,\rho, tA]\,R^*[pb+Kz\,\theta-qb\,\rho, tM2]$$

```
(Xh̃h2 /. {p → pa, q → qa}) (Xh̃h1* /. {p → pb, q → qb}) jin[p1, q1, p2, q2];
tmp = Integrate[%, {p1, -Infinity, +Infinity}, {q1, -Infinity, +Infinity},
    {p2, -Infinity, +Infinity}, {q2, -Infinity, +Infinity}, Assumptions → pa ∈ ℝ];
Collect[tmp⟦1, 2⟧, zD, Simplify];
jh̃21 == ReplacePart[tmp, {1, 2} → %] (* Eq. (C.1i) *)
```

$$j\tilde{h}21 == e^{\frac{i\left(-pa^2+pb^2-qa^2+qb^2\right)zD}{2\,Kz}+i\,(h\,s0+pa\,\Delta x-qa\,\Delta y)}\,jin[pa, qa, pb, qb]\times R[pa, tS]\times R[pa+Kz\,\theta-qa\,\rho, tA]\times R[pa+Kz\,\theta-qa\,\rho, tM2]\,R^*[pb, tM1]\,T^*[pb, tS]\,T^*[-pb-Kz\,\theta+qb\,\rho, tA]$$

Appendix C
calculation of j_{oo}^{ii} and j_{oo}^{ij} , case $p_a = p_b$ and $q_a = q_b$

```
Xõo1 Xõo1* jin[p1, q1, p2, q2];
Jõ11 == Integrate[%, {p1, -Infinity, +Infinity},
  {q1, -Infinity, +Infinity}, {p2, -Infinity, +Infinity},
  {q2, -Infinity, +Infinity}, Assumptions → p ∈ ℝ] (* Eq. (C.2a) *)

Xõo2 Xõo2* jin[p1, q1, p2, q2];
Jõ22 == Integrate[%, {p1, -Infinity, +Infinity},
  {q1, -Infinity, +Infinity}, {p2, -Infinity, +Infinity},
  {q2, -Infinity, +Infinity}, Assumptions → p ∈ ℝ] (* Eq. (C.2b) *)

Xõo1 Xõo2* jin[p1, q1, p2, q2];
Jõ12 == Integrate[%, {p1, -Infinity, +Infinity},
  {q1, -Infinity, +Infinity}, {p2, -Infinity, +Infinity},
  {q2, -Infinity, +Infinity}, Assumptions → p ∈ ℝ] (* Eq. (C.2c) *)

Xõo2 Xõo1* jin[p1, q1, p2, q2];
Jõ21 == Integrate[%, {p1, -Infinity, +Infinity},
  {q1, -Infinity, +Infinity}, {p2, -Infinity, +Infinity},
  {q2, -Infinity, +Infinity}, Assumptions → p ∈ ℝ] (* Eq. (C.2d) *)
Jõ11 == jin[p, q - h ρ, p, q - h ρ] × R[p, tM1] ×
  R[p + Kz Θ - q ρ, tA] × T[p, tS] R*[p, tM1] R*[p + Kz Θ - q ρ, tA] T*[p, tS]

Jõ22 == jin[p, q - h ρ, p, q - h ρ] × R[p, tS] × R[p + Kz Θ - q ρ, tM2] ×
  T[p + Kz Θ - q ρ, tA] R*[p, tS] R*[p + Kz Θ - q ρ, tM2] T*[p + Kz Θ - q ρ, tA]

Jõ12 == e^(-i (h s0+p Δx-q Δy)) jin[p, q - h ρ, p, q - h ρ] × R[p, tM1] ×
  R[p + Kz Θ - q ρ, tA] × T[p, tS] R*[p, tS] R*[p + Kz Θ - q ρ, tM2] T*[p + Kz Θ - q ρ, tA]

Jõ21 == e^(i (h s0+p Δx-q Δy)) jin[p, q - h ρ, p, q - h ρ] × R[p, tS] ×
  R[p + Kz Θ - q ρ, tM2] × T[p + Kz Θ - q ρ, tA] R*[p, tM1] R*[p + Kz Θ - q ρ, tA] T*[p, tS]
```

Appendix C
calculation of j^{ii}_{hh} and j^{ij}_{hh} , case $p_a = p_b$ and $q_a = q_b$

```
Xh̃h1 Xh̃h1* jin[p1, q1, p2, q2];
Jh̃11 == Integrate[%, {p1, -Infinity, +Infinity},
  {q1, -Infinity, +Infinity}, {p2, -Infinity, +Infinity},
  {q2, -Infinity, +Infinity}, Assumptions → p ∈ ℝ] (* Eq. (C.2e) *)

Xh̃h2 Xh̃h2* jin[p1, q1, p2, q2];
Jh̃22 == Integrate[%, {p1, -Infinity, +Infinity},
  {q1, -Infinity, +Infinity}, {p2, -Infinity, +Infinity},
  {q2, -Infinity, +Infinity}, Assumptions → p ∈ ℝ] (* Eq. (C.2f) *)

Xh̃h1 Xh̃h2* jin[p1, q1, p2, q2];
Jh̃12 == Integrate[%, {p1, -Infinity, +Infinity},
  {q1, -Infinity, +Infinity}, {p2, -Infinity, +Infinity},
  {q2, -Infinity, +Infinity}, Assumptions → p ∈ ℝ] (* Eq. (C.2g) *)

Xh̃h2 Xh̃h1* jin[p1, q1, p2, q2];
Jh̃21 == Integrate[%, {p1, -Infinity, +Infinity},
  {q1, -Infinity, +Infinity}, {p2, -Infinity, +Infinity},
  {q2, -Infinity, +Infinity}, Assumptions → p ∈ ℝ] (* Eq. (C.2h) *)

Jh̃11 == jin[p, q, p, q] × R[p, tM1] × T[p, tS] ×
  T[-p - Kz θ + q ρ, tA] R*[p, tM1] T*[p, tS] T*[-p - Kz θ + q ρ, tA]

Jh̃22 == jin[p, q, p, q] × R[p, tS] × R[p + Kz θ - q ρ, tA] ×
  R[p + Kz θ - q ρ, tM2] R*[p, tS] R*[p + Kz θ - q ρ, tA] R*[p + Kz θ - q ρ, tM2]

Jh̃12 == ⅇ^(-ⅈ (h s0+p Δx-q Δy)) jin[p, q, p, q] × R[p, tM1] × T[p, tS] ×
  T[-p - Kz θ + q ρ, tA] R*[p, tS] R*[p + Kz θ - q ρ, tA] R*[p + Kz θ - q ρ, tM2]

Jh̃21 == ⅇ^(ⅈ (h s0+p Δx-q Δy)) jin[p, q, p, q] × R[p, tS] × R[p + Kz θ - q ρ, tA] ×
  R[p + Kz θ - q ρ, tM2] R*[p, tM1] T*[p, tS] T*[-p - Kz θ + q ρ, tA]
```

6.1 moiré fringes

fringe period

```
Clear["Global`*"]; Remove["Global`*"]
jĩn[q1_, q2_] = ⅇ^(-1/8 (q1-q2)^2 w0^2 - 1/4 (q1^2+q2^2) l0^2); (* Eq. (22) *)
Jhh̃11[q1_, q2_] = ⅇ^(-(ⅈ (q1^2-q2^2) zD)/(2 Kz)) jĩn[q1, q2]; (* Eq. (21.a) *)
Jhh̃22[q1_, q2_] = ⅇ^(-(ⅈ (q1^2-q2^2) zD)/(2 Kz) - ⅈ (q1-q2) Δy) jĩn[q1, q2]; (* Eq. (21.b) *)
Jhh̃12[q1_, q2_] = ⅇ^(-(ⅈ (q1^2-q2^2) zD)/(2 Kz) + ⅈ q2 Δy) jĩn[q1, q2]; (* Eq. (21.c) *)
(* inverse Fourier transforms; jo1[y], jo2[y],
and jo12[y] have the same normalising constant *)
Simplify[PowerExpand[InverseFourierTransform[Jhh̃11[q1, q2],
      {q1, q2}, {y, x}, FourierParameters → {1, -1}] /. x → -y]];
Jhh11 == Simplify[Part[%, 1] /. l0^2 + w0^2 → w0^2 /. Kz^2 l0^2 w0^2 + 4 zD^2 → wD^2 Kz^2 l0^2
    (* by using (B2.a) *) /. h → 2 Kz Tan[ΘB]] (* Eq. (23.a) *)
Simplify[PowerExpand[InverseFourierTransform[Jhh̃22[q1, q2],
      {q1, q2}, {y, x}, FourierParameters → {1, -1}] /. x → -y]];
Jhh22 == Simplify[Part[%, 1] /. l0^2 + w0^2 → w0^2 /. Kz^2 l0^2 w0^2 + 4 zD^2 → wD^2 Kz^2 l0^2
    (* by using (B2.a) *) /. h → 2 Kz Tan[ΘB]] (* Eq. (23.b) *)
Jhh12 = Simplify[PowerExpand[InverseFourierTransform[Jhh̃12[q1, q2],
       {q1, q2}, {y, x}, FourierParameters → {1, -1}] /. x → -y]];
(* Jhh12 exponent *)
exponent = Jhh12[[1, 2]] /. 2 Kz^2 l0^2 (l0^2 + w0^2) + 8 zD^2 → 2 wD^2 Kz^2 l0^2;
(* by setting l0^4 = 0 and using (B2.a) *)
(* Jhh12 exponent: real part *)
real =
  FullSimplify[ComplexExpand[Re[exponent]] /. h → 2 Kz Tan[ΘB] /. -(w0^2 Δy^2)/(2 l0^2 wD^2) → -Δy^2/(2 lD^2)];
 (* by using (B2.a) and the θs and θc definitons *)
(* Jhh12 exponent: imaginary part *)
imaginary = ComplexExpand[Im[exponent]];
imaginary = % /. Δy^2 → 0 /. zD → (Kz^2 l0^2 wD^2)/(4 rD)
     (* by using (B2.c) *) /. Δy → 2 (zA2 + tM2 / 2) ρ Tan[ΘB] /. Tan[ΘB] → h/(2 Kz);
Print["Jhh12 = ", Exp[real + I imaginary] (* Eq. (23.c)) *)]
Δρ == (2 π y)/imaginary /. h → 2 π / d (* Eq. (26) *)
```

$$\text{Jhh11} == e^{-\frac{2 y^2}{\text{wD}^2}}$$

$$\text{Jhh22} == e^{-\frac{2 (y-\Delta y)^2}{\text{wD}^2}}$$

$$\text{Jhh12} = e^{-\frac{\Delta y^2}{2\,\text{lD}^2}-\frac{2\,y^2-2\,y\,\Delta y+\Delta y^2}{\text{wD}^2}+\frac{i\,h\,y\left(\frac{\text{tM2}}{2}+\text{zA2}\right)\rho}{\text{rD}}}$$

$$\Lambda\rho == \frac{d\,\text{rD}}{\left(\frac{\text{tM2}}{2}+\text{zA2}\right)\rho}$$

fringe visibility

```
Clear["Global`*"]; Remove["Global`*"]
```

$$\text{Jhh11} = e^{-\frac{2\,y^2}{\text{wD}^2}} \text{(* Eq. (23a) *); Jhh22} = e^{-\frac{2\,(y-\Delta y)^2}{\text{wD}^2}} \text{(* Eq. (23b) *);}$$

$$\text{Jhh12} = e^{-\frac{\Delta y^2}{2\,\text{lD}^2}-\frac{2\,y^2-2\,y\,\Delta y+\Delta y^2}{\text{wD}^2}+\frac{i\,h\,y\left(\frac{\text{tM2}}{2}+\text{zA2}\right)\rho}{\text{rD}}} \text{(* Eq. (23c) *);}$$

$$\text{Print}\left[\text{"}\Gamma = \text{", FullSimplify}\left[\frac{2\,\text{ComplexExpand[Abs[Jhh12]]}}{\text{Jhh11}+\text{Jhh22}}\right]\right] \text{(* Eq. (26) *)}$$

$$\Gamma = e^{-\frac{\Delta y^2}{2\,\text{lD}^2}}\,\text{Sech}\left[\frac{(2\,y-\Delta y)\,\Delta y}{\text{wD}^2}\right]$$

7. results

7.2 Rocking curves symmetries

```
Clear["Global`*"]; Remove["Global`*"];
```

$$\text{R}[\eta\_, \xi\_] = \frac{i\,\text{Sin}\left[\xi\,\sqrt{1+\eta^2}\right]}{\sqrt{1+\eta^2}};$$

```
T[η_, ξ_] = Cos[ξ Sqrt[η^2 + 1]] + η R[η, ξ];
(* zero absorption case:  |T(η,ξ)|^2 = 1 - |R(η,ξ)|^2 *)
R2[η_, ξ_] = ComplexExpand[Abs[R[η, ξ]]]^2;
T2[η_, ξ_] = ComplexExpand[Abs[T[η, ξ]]]^2;
test == Simplify[R2[η, ξ] + T2[η, ξ]]
```

test == 1

The rocking curve symmetry $J_n^{(i)}(-\theta) = J_n^{(i)}(\theta)$ is a consequence of the symmetry $\eta \to -\eta$ of the integrand in (29), provided $\rho = 0$ and the symmetry $\tilde{j}_{\text{in}}(p, q, p, q) = \tilde{j}_{\text{in}}(-p, q, -p, q)$.

We set $\eta_1 = \Delta_e(K_z\,\theta)\tan(\Theta_B)/\pi$.

```
(* change of the integration variables, η = y - η1/2 and η = -y + η1/2 *)
test == {
  Simplify[RRT[y - η1 / 2, η1] - RRT[-y + η1 / 2, -η1]],
  Simplify[TRR[y - η1 / 2, η1] - TRR[-y + η1 / 2, -η1]],
  Simplify[RRR[y - η1 / 2, η1] - RRR[-y + η1 / 2, -η1]],
  Simplify[TRT[y - η1 / 2, η1] - TRT[-y + η1 / 2, -η1]]
 }
test == {0, 0, 0, 0}
```

The identity of the rocking curves TRR and RRT, $J_o^{(1)}(\theta) = J_o^{(2)}(\theta)$, is a consequence of the identity of the integrands in (29) after suitable changes of the integration variable, provided $\rho = 0$ and the symmetry $\tilde{j}_{\text{in}}(p, q, p, q) = \tilde{j}_{\text{in}}(-p, q, -p, q)$. We set $\eta_1 = \Delta_e(K_z\,\theta)\tan(\Theta_B)/\pi$.

```
(* T2 = 1 - R2 *)
RRT[η_, η1_] = R2[η, ξS] × R2[η + η1, ξM] (1 - R2[η + η1, ξS]); (* Jo22 *)
TRR[η_, η1_] = (1 - R2[η, ξS]) R2[η, ξM] × R2[η + η1, ξS]; (* Jo11 *)
RRR[η_, η1_] = R[η, ξS] × R2[η + η1, ξM] × R2[η + η1, ξS]; (* Jh22 *)
TRT[η_, η1_] = (1 - R2[η, ξS]) R2[η, ξM] (1 - R2[-η - η1, ξS]); (* Jh11 *)

(* change of the integration variables, η = y - η1/2 and η = y + η1/2 *)
test == RRT[y - η1 / 2, η1] - TRR[y + η1 / 2, -η1]
test == 0
```

7.3 fringe visibility

```
Clear["Global`*"]; Remove["Global`*"]
(* for the sake of algebraic simplicity, calculations are made for the h state *)
j̃in[q1_, q2_] = e^(-1/8 (q1-q2)^2 w0^2 - 1/4 (q1^2+q2^2) l0^2); (* Eq. (22) *)
Jh̃11[q_] = j̃in[q, q]; (* Eq. (C.2e) *)
Jh̃22[q_] = j̃in[q, q]; (* Eq. (C.2f) *)
Jh̃12[q_] = e^(i q Δy) j̃in[q, q]; (* Eq. (C.2g) *)

Integrate[Jh̃11[q], {q, -Infinity, +Infinity}, Assumptions → w0 > 0 && l0 > 0];
(* Eq. 20.b) *)
Jh = % + Integrate[Jh̃22[q], {q, -Infinity, +Infinity}, Assumptions → w0 > 0 && l0 > 0];
(* Eq. 20.b) *)
Print["Jh = ", Jh]
Ξh = Integrate[Jh̃12[q], {q, -Infinity, +Infinity},
   Assumptions → w0 > 0 && l0 > 0 && Δy ∈ Reals]; (* Eq. 20.c) *)
Print["Ξh = ", Ξh]
Print["Γ = ", PowerExpand[(2 ComplexExpand[Abs[Ξh]]) / Jh] (* Eq. 29 *)]
```

$$Jh = \frac{2\sqrt{2\pi}}{l0}$$

$$\Xi h = \frac{e^{-\frac{\Delta y^2}{2\,l0^2}}\sqrt{2\pi}}{l0}$$

$$\Gamma = e^{-\frac{\Delta y^2}{2\,l0^2}}$$

Appendix B - free-space propagation

propagation of the single particle wave function (Gaussian model)

```
Clear["Global`*"]; Remove["Global`*"];
(* Gaussian wave packet; reciprocal-space representation;
p and l0 are measured orthogonally to the optical axis z *)
ψ̃in[p_] = e^(-p^2 l0^2/4);
(* free-space propagator; z is measured along the propagation axis;
pm = +1 if propagation is along Ko;
pm = -1 if propagation is along Kh;
pm = 0 if p → q and x → y *)
F[p_, z_] = e^(-i p^2 z/(2 Kz)) e^(I pm p z Tan[ΘB]);
(* free-space propagation *)
InverseFourierTransform[F[p, z] ψ̃in[p], p, x, FourierParameters → {0, -1}];
Print["ψ̃in[x;z] ∝ ", Part[%, 2]]
```

$\tilde{\psi}_{in}[x;z] \propto e^{-\frac{Kz\,(x+pm\,z\,Tan[\Theta B])^2}{Kz\,l0^2+2\,i\,z}}$

$\tilde{\psi}_{in}[x;\,z] \propto e^{-\frac{(x+pm\,z\,Tan[\Theta B])^2}{lz^2}+\frac{I\,Kz\,(x+pm\,z\,Tan[\Theta B])^2}{2\,rz}}$

lz : beam radius

```
Simplify[ComplexExpand[Re[-Kz (x + pm z Tan[ΘB])^2/(Kz l0^2 + 2 i z)]]];
lz^2 == Expand[Expand[-(x + pm z Tan[ΘB])^2/%] /. 4/(Kz^2 l0^2) → Tan[θs]^2]
 (* Eq. (B.1a), Tan[θs] definition *)
```

$lz^2 == l0^2 + z^2\,Tan[\theta s]^2$

$\tilde{\psi}_{in}[x;\,z] \propto e^{-\frac{(x+pm\,z\,Tan[\Theta B])^2}{lz^2}+\frac{I\,Kz\,(x+pm\,z\,Tan[\Theta B])^2}{2\,rz}}$

rz : wavefront radius of curvature

$$\text{Simplify}\left[\text{ComplexExpand}\left[\text{Im}\left[-\frac{\text{Kz}\ (\text{x} + \text{pm z Tan}[\Theta\text{B}])^2}{\text{Kz } \text{l0}^2 + 2\, i\, \text{z}}\right]\right]\right];$$

$$\text{Expand}\left[\frac{\text{Kz}\ (\text{x} + \text{pm z Tan}[\Theta\text{B}])^2}{2 \times \%}\right]\ /.\ \frac{\text{Kz}^2\ \text{l0}^4}{4} \to \frac{\text{l0}^2}{\text{Tan}[\theta\text{s}]^2}$$

(* Eq. (B.1b), Tan[θs] definition *);

Print["r_z = ", %]

$$\text{Simplify}\left[\text{ComplexExpand}\left[\text{Im}\left[-\frac{\text{Kz}\ (\text{x} + \text{pm z Tan}[\Theta\text{B}])^2}{\text{Kz } \text{l0}^2 + 2\, i\, \text{z}}\right]\right]\right];$$

$$\text{Together}\left[\frac{\text{Kz}\ (\text{x} + \text{pm z Tan}[\Theta\text{B}])^2}{2 \times \%}\right]\ /.\ \text{Kz}^2\ \text{l0}^4 + 4\, \text{z}^2 \to \text{Kz}^2\ \text{l0}^2\ \text{lz}^2$$

(* by the Tan[θs] and lz definitions *);

Print["r_z = ", %]

$$r_z = z + \frac{\text{l0}^2\ \text{Cot}[\theta\text{s}]^2}{z}$$

$$r_z = \frac{\text{Kz}^2\ \text{l0}^2\ \text{lz}^2}{4\, z}$$

propagation of the density matrix (Gauss Schell-model)

Clear["Global`*"]; Remove["Global`*"]

$$\tilde{\text{j}}_{\text{in}}[\text{q1\_}, \text{q2\_}] = e^{-\frac{1}{8}\ (\text{q1}-\text{q2})^2\ \text{w0}^2 - \frac{1}{4}\ \left(\text{q1}^2+\text{q2}^2\right)\ \text{l0}^2};$$

$$\text{F}[\text{q\_}, \text{z\_}] = e^{-\frac{i\, \text{q}^2\, \text{z}}{2\, \text{Kz}}};$$

(* propagation *)

$$\tilde{\text{j}}_{\text{z}}[\text{q1\_}, \text{q2\_}] = \text{F}[\text{q1}, \text{z}]\ \tilde{\text{j}}_{\text{in}}[\text{q1}, \text{q2}]\ (\text{Conjugate}[\text{F}[\text{q2}, \text{z}]]\ /.\ \text{Conjugate}[\text{a\_}] \to \text{a});$$

(* transformation back to the direct space *)

$$\text{Part}\left[\text{InverseFourierTransform}\left[\tilde{\text{j}}_{\text{z}}[\text{q1}, \text{q2}],\ \{\text{q1}, \text{q2}\}, \{\text{y1}, \text{x}\}, \text{FourierParameters} \to \{1, -1\}\right]\ /.\ \text{x} \to -\text{y2}, 1\right];$$

$$\text{j}_{\text{z}}[\text{y1\_}, \text{y2\_}] = \%\ /.\ \text{l0}^2 + \text{w0}^2 \to \text{w0}^2; (\text{* lo << w0 *})$$

Print["j_z[y1,y2] = ", j_z[y1, y2]]

$$\text{j}_{\text{z}}[\text{y1},\text{y2}] = e^{-\frac{\text{Kz}\ \left(\text{Kz w0}^2\ (\text{y1}-\text{y2})^2 + 2\, \text{Kz l0}^2\ \left(\text{y1}^2+\text{y2}^2\right) + 4\, i\ \left(-\text{y1}^2+\text{y2}^2\right)\ \text{z}\right)}{2\, \text{Kz}^2\ \text{l0}^2\ \text{w0}^2 + 8\, \text{z}^2}}$$

beam radius and correlation length

```
Simplify[ComplexExpand[Re[Part[jz[y1, y2], 2]]]];
tmp = Collect[%, (y1 - y2)^2, Simplify];
Print["exponent: real part = ", %]
-Simplify[1/Coefficient[tmp, y1^2 + y2^2]] /. 4/(Kz^2 l0^2) → Tan[θs]^2
  (* Eq. (B.1c), θs definition *);
Print["wz^2 = ", %](* Eq. (B.2a) *)
-Simplify[1/(2 Coefficient[tmp, (y1 - y2)^2])] /. 4/(Kz^2 w0^2) → Tan[θc]^2
  (* Eq. (B.2d), θc definition *);
Print["lz^2 = ", %](* Eq. (B.2b) *)
```

exponent: real part = $-\frac{Kz^2\, l0^2\, \left(y1^2 + y2^2\right)}{Kz^2\, l0^2\, w0^2 + 4\, z^2} - \frac{Kz^2\, w0^2\, (y1 - y2)^2}{2\, Kz^2\, l0^2\, w0^2 + 8\, z^2}$

$w_z^2 = w0^2 + z^2\, \mathrm{Tan}[\theta s]^2$

$l_z^2 = l0^2 + z^2\, \mathrm{Tan}[\theta c]^2$

curvature radius

```
tmp = Simplify[ComplexExpand[Im[Part[jz[y1, y2], 2]]]];
Print["imaginary part = ", %]
Expand[Kz (y1^2 - y2^2)/(2 tmp)] /. Kz^2 w0^2/4 → 1/Tan[θc]^2 (* θc definition, Eq. (B.2d) *);
Print["rz = ", %](* Eq. (B.2c) *)
Together[Kz (y1^2 - y2^2)/(2 tmp)] /. Kz^2 l0^2 w0^2 + 4 z^2 → Kz^2 l0^2 wz^2
  (* by the Tan[θs] and lz definitions *);
Print["rz = ", %](* Eq. (B.2c) *)
```

imaginary part = $\frac{2\, Kz\, \left(y1^2 - y2^2\right) z}{Kz^2\, l0^2\, w0^2 + 4\, z^2}$

$r_z = z + \frac{l0^2\, \mathrm{Cot}[\theta c]^2}{z}$

$r_z = \frac{Kz^2\, l0^2\, wz^2}{4\, z}$

Appendix D - shear interferometry

interference of parallel Gaussian beams

```
Clear["Global`*"]; Remove["Global`*"];
```

$\phi 1 = e^{-\frac{y^2}{lz^2}+\frac{i\,Kz\,y^2}{2\,rz}};$

$\phi 2 = e^{-\frac{(y-\Delta y)^2}{lz^2}+\frac{i\,Kz\,(y-\Delta y)^2}{2\,rz}};$

```
ϕ1 FullSimplify[ComplexExpand[Conjugate[ϕ2]]]
Ξ = Integrate[%, {y, -Infinity, +Infinity},
   Assumptions → lz > 0 && rz > 0 && Δy > 0 && Kz > 0];
Print["Ξ = ", Ξ]
```

$J1 = \text{Integrate}\left[e^{-\frac{2y^2}{lz^2}}, \{y, -\text{Infinity}, +\text{Infinity}\}, \text{Assumptions} \to lz > 0\right];$

```
Print["J1 = ", J1]
```

$J2 = \text{Integrate}\left[e^{-\frac{2(y-\Delta y)^2}{lz^2}}, \{y, -\text{Infinity}, +\text{Infinity}\}, \text{Assumptions} \to lz > 0 \,\&\&\, x0 > 0\right];$

```
Print["J2 = ", J2]
```

$\Gamma = \frac{2\,\Xi}{J1+J2}$ (* visibility *); Print["Γ = ", Γ]

```
(* by using (B1-a), (B1-b), and (B1-c) *)
```

$\text{Simplify}\left[\Gamma[[2]] \;//.\; \left\{lz \to \text{Sqrt}\left[l0^2 + z^2\,\text{Tan}[\theta s]^2\right],\right.\right.$

$\left.\left. rz \to z + \frac{l0^2\,\text{Cot}[\theta s]^2}{z},\ \text{Tan}[\theta s] \to \frac{2}{Kz\,l0},\ \text{Cot}[\theta s] \to \frac{Kz\,l0}{2}\right\}\right];$

```
Print["Γ = ", Exp[%]]
```

$e^{-\frac{y^2}{lz^2}+\frac{i\,Kz\,y^2}{2\,rz}-\frac{i\,(Kz\,lz^2-2\,i\,rz)\,(y-\Delta y)^2}{2\,lz^2\,rz}}$

$\Xi = e^{-\frac{(Kz^2\,lz^4+4\,rz^2)\,\Delta y^2}{8\,lz^2\,rz^2}}\;lz\sqrt{\frac{\pi}{2}}$

$J1 = lz\sqrt{\frac{\pi}{2}}$

$J2 = lz\sqrt{\frac{\pi}{2}}$

$\Gamma = e^{-\frac{(Kz^2\,lz^4+4\,rz^2)\,\Delta y^2}{8\,lz^2\,rz^2}}$

$\Gamma = e^{-\frac{\Delta y^2}{2\,l0^2}}$

interference of intersecting Gaussian beams

```
Clear["Global`*"]; Remove["Global`*"];
```

$\phi 1 = e^{-\frac{y^2}{lz^2}+\frac{i\,Kz\,y^2}{2\,rz}};$

$\phi 2 = e^{-\frac{y^2}{lz^2}+\frac{i\,Kz\,y^2}{2\,rz}+i\,Kz\,\rho 0\,y};$

```
Ξ = Integrate[ϕ1 FullSimplify[ComplexExpand[Conjugate[ϕ2]]],
   {y, -Infinity, +Infinity}, Assumptions → lz > 0 && rz > 0 && Kz > 0 && ρ0 > 0];
Print["Ξ = ", Ξ]
```

$J1 = \text{Integrate}\left[e^{-\frac{2\,y^2}{lz^2}}, \{y, -\text{Infinity}, +\text{Infinity}\}, \text{Assumptions} \to lz > 0\right];$

```
Print["J2 = J1 = ", J1]
J2 = J1;
Γ = 2 Ξ / (J1 + J2) (* visibility *);
Print["Γ = ", Γ]
```

$\Xi = e^{-\frac{1}{8} Kz^2\, lz^2\, \rho 0^2}\, lz \sqrt{\frac{\pi}{2}}$

$J2 = J1 = lz \sqrt{\frac{\pi}{2}}$

$\Gamma = e^{-\frac{1}{8} Kz^2\, lz^2\, \rho 0^2}$
